# Effects of exogenous SLs on growth and physiological characteristics of flue-cured tobacco seedlings under different degrees of drought stress

**DOI:** 10.3389/fpls.2024.1473565

**Published:** 2025-01-20

**Authors:** Xiao-dong Wang, Yi-nan Zhang, Xiao-guo Wang, Ye Zhuang, Shao-hua Ge

**Affiliations:** ^1^ College of Agriculture, Henan University of Science and Technology, Luoyang, Henan, China; ^2^ Production Technology Section, Henan Province Tobacco Company, Jiyuan, Henan, China; ^3^ Guizhou Tobacco Company Qiandongnan Branch Tobacco Technology Center, Guizhou Tobacco Company Qiandongnan Prefecture Company, Guizhou, China

**Keywords:** drought stress, tobacco seedling, SLs, physiological response, antioxidant

## Abstract

**Background:**

Drought stress severely affects global crop yields, reduces water availability, and hinders growth. Strigolactones can alleviate damage caused by various abiotic stresses in plants; however, limited research has been conducted on their ability to enhance drought tolerance in tobacco.

**Methods:**

This study evaluated the drought tolerance of ‘Qin Tobacco 96’ (drought-tolerant) and ‘Yun Tobacco 116’ (moisture-sensitive) before and after the application of gibberellic acid lactone at a concentration of 0.2 mg·L⁻¹ under three drought conditions: mild, moderate, and severe. The primary drought tolerance traits were identified from 29 related indicators, including agronomic traits, photosynthetic efficiency, reactive oxygen metabolism, antioxidant enzyme activities, osmotic regulators, and hormone regulation, using affiliation function, principal component analysis, and cluster analysis to categorize the traits. The degree of drought tolerance enhancement in the two tobacco varieties was evaluated under various treatments.

**Results:**

Spraying exogenous strigolactones reduced the adverse effects of drought stress, particularly in the moisture-sensitive Y116 variety. Under drought stress, chlorophyll content and photosynthetic parameters significantly decreased, whereas strigolactone treatment increased both chlorophyll content and photosynthetic efficiency. Strigolactones reduced the accumulation of reactive oxygen species and malondialdehyde content, enhancing the antioxidant capacity of both varieties. Additionally, strigolactones increased the levels of osmoregulatory substances and activated the production of antioxidant enzymes, thereby enhancing drought tolerance. Furthermore, drought stress disrupted the balance of endogenous hormones, decreasing levels of auxin, gibberellic acid, and ribosylzeatin, while increasing abscisic acid levels. Exogenous strigolactones restored this hormonal balance.

**Conclusion:**

Sixteen traits associated with drought tolerance in tobacco were analyzed using principal component analysis, the traits were classified using cluster analysis, and the magnitude of the D-value was determined by calculating the values of the affiliation function and their respective weights. The results indicated that a concentration of 0.2 mg·L⁻¹ of strigolactones enhanced the drought tolerance of tobacco across different levels of drought stress and promoted the growth and development of flue-cured tobacco. However, the interactions between strigolactones and various hormones under drought stress require further investigation to elucidate the underlying molecular mechanisms. The application methods of strigolactones should be optimized.

## 1 Introduction

Drought stress, a significant abiotic factor, has a significant impact on global crop productivity (Anderegg et al., 2013). Approximately one-third of the global arable land is affected by drought stress to varying degrees over extended periods, with even non-arid regions being susceptible to unpredictable climate and environmental changes that lead to such stress (LeNoble et al., 2004; Ort et al., 2015; Huang et al., 2023). The annual increase in water scarcity is expanding arid regions worldwide, thereby is exacerbating drought conditions (Zhou et al., 2015; Gelaw et al., 2023). Therefore, it is imperative to urgently address drought. Drought stress poses a significant threat to crop growth, resulting in persistent delays and stagnation in agricultural production and hindering the sustainable advancement of modern agriculture. Drought stress reduces water availability in crop environments, leads to underdeveloped roots, curled leaves, yellowing, wilting, and delayed plant growth (Zhou et al., 2015; Li et al., 2018; Sun et al., 2022). In severe cases, premature wilting and plant death may occur. Dry matter accumulation in crops serves as a critical indicator of organic matter accumulation and nutrient content (Anderegg et al., 2013). Photosynthesis, which is essential for normal plant growth, development, and biomass accumulation, is significantly inhibited by drought stress (Malliarakis et al., 2015; Ye et al., 2016). This inhibition occurs as the water potential that safeguards stomatal cells decreases, resulting in stomatal closure and impaired carbon dioxide absorption and utilization by the plant (Iqbal et al., 2015). Subsequent stomatal closure increases intercellular carbon dioxide concentration, which damages chloroplast structure, leading to the disintegration of thylakoids and disruption of PSII, ultimately decreasing the photosynthetic performance of mesophyll cells (Fernández-San Millán et al., 2018). When plants lose their self-regulatory capacity due to severe drought stress, the reactive oxygen species (ROS) system exhibits metabolic dysregulation and even sustains damage, producing substantial ROS (del Rio et al., 2006). This leads to membrane lipid peroxidation, impairs normal cell membrane function and results in the generation of the peroxidation product malondialdehyde (MDA) (Guo et al., 2006; Leitao and Enguita, 2016). Antioxidant enzymes, essential for plant self-protection during stress, function to mitigate stress levels. The activities of superoxide dismutase (SOD), peroxidase (POD), and catalase (CAT) fluctuate under stress. However, under severe stress, the function of antioxidant enzymes is compromised, hindering the clearance of intracellular peroxidation products and ultimately leading to cell damage that impacts normal physiological and metabolic functions (Xia et al., 2023). Furthermore, the accumulation of substances within plant cells stabilizes the chemical compounds that regulate cell permeability. In response to drought stress, numerous regulatory compounds are produced, increasing the concentration of cell fluids and enhancing protoplast hydrophilicity, which improves plant drought resistance (Mohammadi et al., 2017; Yang et al., 2023). Concurrently, Drought stress activates key enzymes involved in plant carbon and nitrogen metabolism, thereby maintain normal cellular metabolism and alters the composition of cellular osmolytes. Under these conditions, the levels of plant endogenous hormones decrease, negatively impacts normal physiological metabolism (Gupta and Rashotte, 2014). The complex regulatory system results from the interactions of various endogenous hormones regulate diverse metabolic processes related to plant growth and development.

In recent years, numerous studies have investigated the mechanisms through which formulations, including plant hormones, amino acids, antioxidants, micronutrients, organic acids, plant extracts, and nanomaterials, alleviate abiotic stress. Strigolactones (SLs), recognized as a classic plant hormone, play a crucial role in numerous physiological and metabolic processes in plants. These include functioning as a rhizosphere signaling molecule, inducing hyphal branching of arbuscular mycorrhizal fungi, and regulating leaf age, thereby influencing plant growth and developmen (Kisugi et al., 2013; Sang et al., 2014; Zwanenburg et al., 2016). SLs emerge as a central focus in numerous studies regarding their role in helping plants adapt to environmental stress, SLs can modulate osmotic pressure, thereby assisting plants in maintaining water balance (Li et al., 2022). Under nutrient stress, SLs can also assist plants in managing water loss by regulating root growth (Yoneyama and Brewer, 2021). In both biotic and abiotic stress conditions, SLs can function as significant regulatory substances (Guercio et al., 2023). Under both biotic and abiotic stress, SLs can act as significant regulatory substances (Wen et al., 2024). Raja et al. propose that SLs improve chromium tolerance in tomatoes by reducing the excess ROS produced in response to chromium toxicity (Raja et al., 2023). Scaffidi et al. propose that SLs play a critical role in modulating plant responses to abiotic stresses, including drought (Scaffidi et al., 2014). SLs can enhance nutrient absorption through multiple mechanisms, including the regulation of root architecture and interactions with other hormones (Al-Babili and Bouwmeester, 2015).

Tobacco growth and development depend on adequate water content, as sufficient moisture is essential for producing high-quality tobacco leaves and maximizing yield (Hansch et al., 2001; Fernández-San Millán et al., 2018) Although studies on SLs enhance plant stress tolerance are relatively common, research on the application of exogenous SLs—especially their role in improving drought resistance in flue-cured tobacco—remains limited. Given the diverse mechanisms through which SLs enhance stress resistance, this study aims to systematically investigate various indicators by applying exogenous SLs to flue-cured tobacco. The indicators examined include agronomic traits, photosynthetic fluorescence systems, reactive oxygen metabolism, antioxidant enzyme activities, osmotic regulatory substances, and hormone interactions. We employ principal component analysis and affiliation function analysis to evaluate drought tolerance in tobacco. This approach not only aids in speculating on the enhancement of physiological responses related to internal drought resistance in flue-cured tobacco but also further elucidates its underlying molecular mechanisms. Furthermore, it will evaluate whether SLs can enhance the productivity of flue-cured tobacco, thereby providing a theoretical foundation for drought prevention in field production practices.

## 2 Materials and methods

### 2.1 Test materials

In this study, two tobacco varieties, namely ‘Qinyan 96’ (a drought-tolerant type) and ‘Yunyan 116’ (a water-sensitive type), were tested. The strigolactone (SLs) used in the study was provided by the Beijing Solarbio Science &Technology Co., Ltd.

### 2.2 Experimental design and management

The experiment utilized two tobacco varieties, ‘Q96’ and ‘Y116’, to facilitate a comparative analysis of their responses to drought stress. Based on extensive preliminary research and trials, it was determined that applying 0.2 mg•L⁻¹ of SLs yielded favorable results (The manuscript containing this concentration screening data is currently being submitted for publication); therefore, this concentration was selected for the experiment.

There were seven treatments:

1. CK: Adequate water supply (75–80% soil water holding capacity)
2. D1: Light drought (55–60% soil water holding capacity)
3. D2: Moderate drought (45–50% soil water holding capacity)
4. D3: Severe drought (35–40% soil water holding capacity)
5. T1: Light drought (55–60% soil water holding capacity) + 0.2 mg•L⁻¹ SLs
6. T2: Moderate drought (45–50% soil water holding capacity) + 0.2 mg•L⁻¹ SLs
7. T3: Severe drought (35–40% soil water holding capacity) + 0.2 mg•L⁻¹ SLs

Each treatment was replicated ten times, with each tobacco seedling transplanted into plastic pots of approximately 30 L in volume, resulting in a total of 70 pots. Each pot contained 20 kg of soil, with an application of 3.5 g of pure nitrogen, maintaining a nitrogen:phosphorus(N:P:K) ratio of 1:1.5:1. All pots were placed in a drought shelter at the experimental farm of Henan University of Science and Technology, with the roof covered by sealed sunlight panels that have a light transmittance exceeding 90%. A tensiometer was installed in each pot to measure the relative soil moisture content, which demonstrated a highly significant negative correlation with the tensiometer readings (correlation coefficient r = -0.921**). The drought stress treatment continued for 15 days, commencing from the end of the transplanting period (5 days post-transplant). At the conclusion of the drought stress period, all treatments, with the exception of the control group, were sprayed with SLs. All parameters were recorded on the 15th day of drought stress and again on the 15th day following SLs application, with samples collected for measurement. The specific experimental treatment settings are outlined in **Table 1**.

**Table 1:** Test processing settings.

Variety	Treatments code	Treatments	
		Soil water holding capacity (%)	SLs concentration(mg.L^-1^)
Q96 Y116	CK	75∼80	0
	D1	55∼60	0
	D2	45∼50	0
	D3	35∼40	0
	T1	55∼60	0.2
	T2	45∼50	0.2
	T3	35∼40	0.2

CK: Adequate water supply (75–80% soil water holding capacity), D1: Light drought (55–60% soil water holding capacity), D2: Moderate drought (45–50% soil water holding capacity), D3: Severe drought (35–40% soil water holding capacity), T1: Light drought (55–60% soil water holding capacity) + 0.2 mg.L^-1^SLs, T2: Moderate drought (45–50% soil water holding capacity) + 0.2 mg.L^-1^SLs, T3: Severe drought (35–40% soil water holding capacity) + 0.2 mg.L^-1^SLs.

### 2.3 Measurement index and methods

On the 15th day of drought stress, as well as 15 days post-application of SLs, three tobacco plants exhibiting similar growth vigor were selected from each treatment for the assessment of their physiological traits. Concurrently, fresh leaves from the same position at the top of the plants were collected and stored at -80°C for subsequent measurements.

#### 2.3.1 Measuring agronomic traits

The plant height, maximum leaf length, maximum leaf width, and maximum leaf area of tobacco seedlings in each treatment group were measured using a ruler. Subsequently, the tobacco samples were placed in an oven, initially blanched at 105°C for 30 minutes, followed by drying at 70°C until a constant weight was achieved. Finally, the dry weight per plant was measured using an electronic balance.


(1) $$\begin{array}{c} \text{Maximum leaf area}=\\ \text{Maximum leaf length}\times \text{Maximum leaf width}\times 0.6345 \end{array}$$

#### 2.3.2 Determination of chlorophyll content

The third leaf beneath the core leaf of the tobacco plant was selected, and the tobacco leaves were purified with acetone. The absorbance of chlorophyll a and chlorophyll b was measured at wavelengths of 663 nm and 645 nm, respectively, using a spectrophotometer for chlorophyll content analysis (Giokas et al., 2011).

#### 2.3.3 Measurement of photosynthetic parameters

Between 9:00 and 11:00 on a clear morning, the same functional leaves of tobacco plants were measured using the Li-6400 XT portable photosynthesis system to assess photosynthetic parameters. These parameters included photosynthetic rates (P_n_), stomatal conduction (G_s_), transpiration rate (T_r_), and substomatal cavity CO_2_ concentration (C_i_) (Iqbal et al., 2015).

#### 2.3.4 Determination of chlorophyll fluorescence parameters

Chlorophyll fluorescence parameters were measured between 9:00 am and 11:00 am on a sunny day using PAM-2100 portable modulated fluorometer (Walz Company, Germany) (van der Tol et al., 2009). The following formulae were used for calculation:


(2) Fv/Fm=(Fm−F0)/Fm

(3) PSII=(Fm′−Fs)/Fm′

(4) qP=(Fm′−Fs)/(Fm′−F0′)

(5) NPQ=(Fm−Fm′)/Fm"

Where: F0 is the minimum fluorescence measured in the dark-adapted state, Fm is the maximum fluorescence measured under fully saturating light conditions; Fm′ is the maximum fluorescence achieved under light conditions, Fs is the steady-state fluorescence under light; F0′ is the minimum fluorescence under light conditions.

#### 2.3.5 Measurement of O_2_^-^ generation rate

Fluorescent probes were employed to monitor changes in O_2_^-^ concentration during redox reactions, allowing for the detection of the generation rate (Begum et al., 2020).

#### 2.3.6 Determination of malondialdehyde (MDA) content

The sample was reacted with thiobarbituric acid to generate a red complex, and the absorbance was measured at 532 nm to quantify the malondialdehyde (MDA) content using a standard curve (Hafez et al., 2020).

#### 2.3.7 Measurement of antioxidant enzyme activity

Superoxide dismutase (SOD) activity was assessed using the nitroblue tetrazolium photochemical reduction method, with 560 nm selected as the measurement wavelength (Mohamed et al., 2015). The guaiacol method for determining peroxidase (POD) uses guaiacol as a substrate, with the enzyme catalyzing its oxidation reaction to produce a color change. Absorbance is then measured spectrophotometrically at approximately 470 nm to calculate enzyme activity (Surowsky et al., 2013; Batool et al., 2020). Catalase (CAT) activity was assessed by measuring the change in concentration of hydrogen peroxide in the reaction mixture at 240 nm (Batool et al., 2020).

#### 2.3.8 Determination of osmotic regulation substances content

For the determination of proline (Pro) by the acid ninhydrin method, the absorbance of the sample reacting with hydrochloric acid and ninhydrin to form a purple compound was measured at 570 nm using a colorimeter to calculate the Pro concentration. In the ninhydrin colorimetric method, 420 nm was chosen as the determination wavelength to measure the absorbance of the ninhydrin-sugar complex to accurately measure the soluble sugar content (Yu et al., 2022).

#### 2.3.9 Measurement of carbon and nitrogen metabolism enzyme activity

Nitrate reductase (NR) (Hansch et al., 2001) and 1, 5-diphosphate ribulose-carboxylase (Rubisco) (Suganami et al., 2020) were detected using their respective kits (Solebao Biotechnology Co., LTD.), with the concentration calculated from the absorption value at 450 nm wavelength using an enzyme marker.

#### 2.3.10 Determination of hormone content

The ELISA method (Solebao Biotechnology Co., LTD.) was employed to test the auxin (IAA), gibberellic acid (GA), ribosylzeatin (ZR), and abscisic acid (ABA) contents in tobacco leaves (Xing et al., 2023).

### 2.4 Data processing

#### 2.4.1 Indicator analysis

Data processing and analysis utilized statistical software Excel and SPSS. One-way ANOVA and LSD tested the differences between various water treatments in the same variety.Correlation, principal component, and cluster analyses were conducted for 29 traits. Origin 2021 software was used for mapping.

#### 2.4.2 Comprehensive evaluation of drought effectiveness

To assess the effect of spraying SLs on improving drought tolerance in tobacco, the initial step involved calculating the affiliation function values for each composite indicator to facilitate effective comparison. Subsequently, principal components were extracted, and the variance contribution ratio was derived through principal component analysis (PCA). This contribution ratio was then employed to calculate the principal component weights for each indicator. Finally, the composite evaluation value (D-value) is calculated by multiplying and summing the affiliation function values with the principal component weights. The specific formula is as follows:


(6) $$\text{U}({\text{X}}_{\text{j}})=({\text{X}}_{\text{j}}-{\text{X}}_{\min})/({\text{X}}_{\max}-{\text{X}}_{\min})$$

(7) $${\text{W}}_{\text{j}}={\text{P}}_{\text{j}}/\Sigma {\text{P}}_{\text{j}}\text{, j}=1,\;2,\;3,\ldots ,\text{ n}$$

(8) $$\text{D}=\Sigma [\text{U}({\text{X}}_{\text{j}})\times {\text{W}}_{\text{j}}]\text{, j}=1,\;2,\;3,\ldots ,\text{ n}$$

In this context, X_j_ denotes the affiliation function value of each treatment for the j^th^ index, while X_max_ and X_min_ represent the highest and lowest values of this index under different SL treatments, respectively. The weight of the j^th^ principal component is denoted as W_j_, and P_j_ represents its variance contribution ratio. The composite evaluation value D reflects each treatment’s drought tolerance under drought conditions, ranging from 0.00 to 1.00, where higher scores indicate greater improvement in drought tolerance.

## 3 Results

### 3.1 Effects of exogenous SLs on growth of flue-cured tobacco seedlings under different degrees of drought stress

#### 3.1.1 Plant height parameters

**Figure 1:**
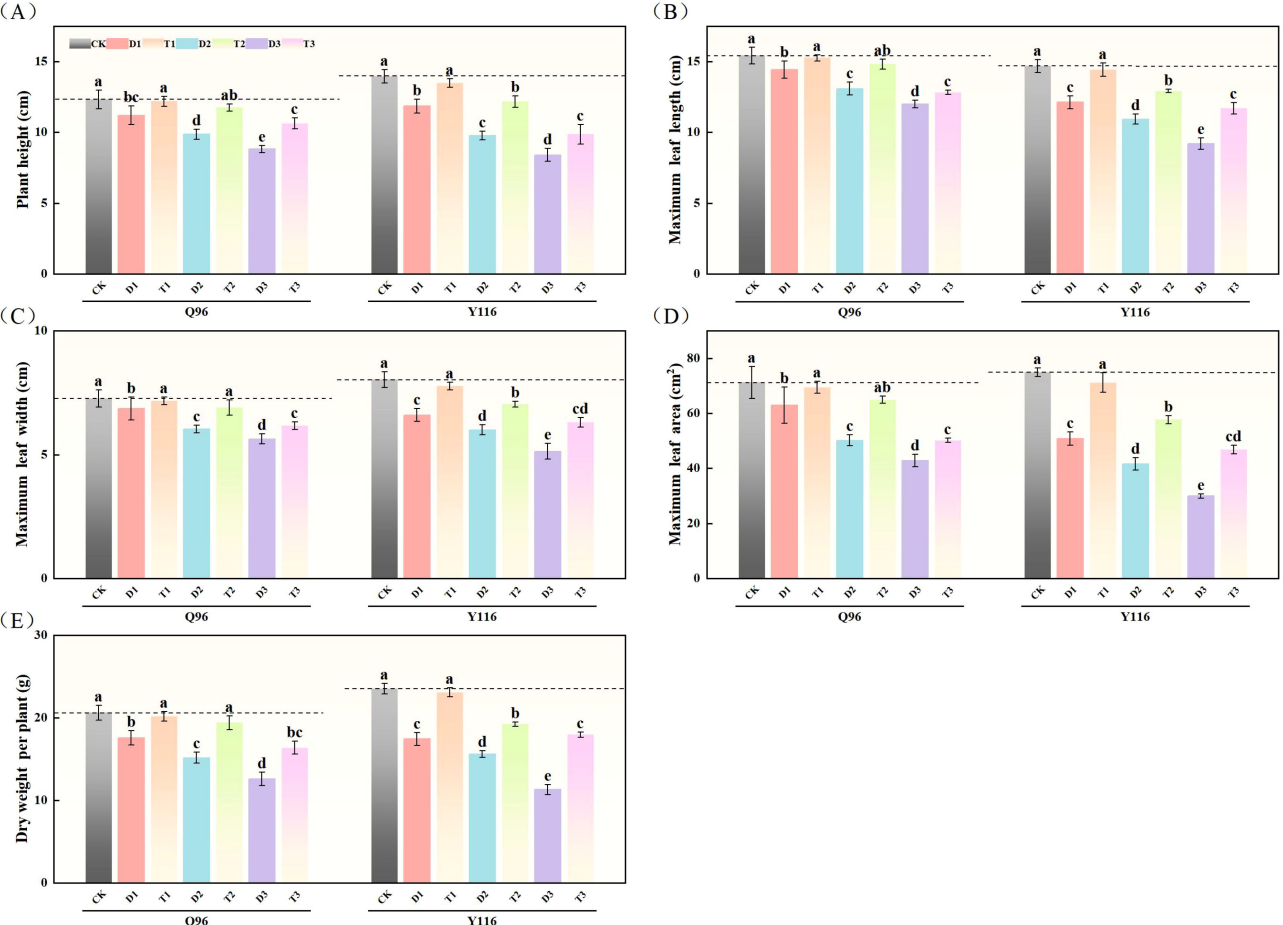
Effect of exogenous SLs on growth of flue-cured tobacco seedlings under different drought stress. **(A)** Effects of different drought stress on plant height of flue-cured tobacco seedlings, **(B)** Effects of different drought stress on maximum leaf length of flue-cured tobacco seedlings, **(C)** Effects of different drought stress on maximum leaf width of flue-cured tobacco seedling. **(D)** Effects of different drought stress on maximum leaf area of flue-cured tobacco seedlings, **(E)** Effects of different drought stress on dry weight per plant of flue-cured tobacco seedlings. The error bars represent the standard error for each group, and different letters (a, b, c, d, e) indicate significant differences (p< 0.05). “Q96” and “Y116” mean flue-cured tobacco varieties Qinyan 96 and Yunyan 116, respectively. CK, D1, D2, D3, T1, T2,and T3 represent adequate water supply (75–80% soil water holding capacity), light drought (55–60% soil water holding capacity), moderate stress (45–50% soil water holding capacity) and severe drought (35–40% soil water holding capacity), light drought (55–60% soil water holding capacity) + 0.2 mg.L^-1^SLs, moderate stress (45–50% soil water holding capacity) + 0.2 mg.L^-1^SLs and severe drought (35–40% soil water holding capacity) + 0.2 mg.L^-1^SLs.

As drought stress intensified, the plant height of the Q96 and Y116 varieties gradually decreased. In comparison to the control group (CK) under drought stress, the plant height of tobacco (**Figure 1A**) significantly decreased under the D1, D2, and D3 treatments, with Q96 decreasing by 9.2%, 20.0%, and 28.4%, respectively, and Y116 decreasing by 15.0%, 30.1%, and 39.9%, respectively. There was no significant change in the plant height of T1 compared to CK following the application of SLs. Compared to D1, D2, and D3, the plant height significantly increased following the application of SLs under the same drought conditions, with Q96 increasing by 8.93%, 19.3%, and 20.4%, respectively, and Y116 increasing by 13.8%, 24.6%, and 17.5%, respectively.

#### 3.1.2 Maximum leaf length parameters

**Figure 1B** shows that the maximum leaf length of tobacco increased with the intensification of drought in the Q96 and Y116 varieties. Compared to the control group (CK), the maximum leaf length of Q96 decreased by 6.5%, 15.1%, and 22.3% under D1, D2, and D3 treatments, respectively, while Y116 exhibited significant decreases of 17.5%, 25.6%, and 37.4%. Compared to D1, D2, and D3, the maximum leaf length of T1, T2, and T3 increased by 5.8%, 13.2%, and 6.9% in Q96, and by 19.00%, 18.3%, and 27.2% in Y116, respectively.

#### 3.1.3 Maximum leaf width parameters

Both tobacco varieties exhibited a reduction in maximum leaf width as a result of drought (**Figure 1C**). In comparison to the control group (CK), Q96 decreased significantly by 5.5%, 17.00%, and 22.5% under D1, D2, and D3 treatments, respectively, while Y116 decreased by 17.8%, 25.3%, and 36.1%. The application of SLs resulted in an increase in maximum leaf width for T1, T2, and T3 compared to D1, D2, and D3, with Q96 increasing by 4.4%, 14.4%, and 9.5%, respectively, and Y116 increasing by 17.7%, 17.2%, and 22.7%.

#### 3.1.4 Maximum leaf area parameters

Drought stress significantly reduced the maximum leaf area of both tobacco varieties (**Figure 1D**). In comparison to the control group (CK), Q96 decreased by 11.6%, 29.6%, and 39.8% under D1, D2, and D3 treatments, respectively, while Y116 decreased by 32.2%, 44.4%, and 60.0%. Following the application of SLs, the maximum leaf area of T1, T2, and T3 increased compared to D1, D2, and D3, with Q96 increasing by 10.2%, 29.4%, and 17.0%, respectively, and Y116 increasing by 40.1%,38.6%, and 56.3%.

#### 3.1.5 Dry weight per plant parameters

The dry weight per plant of tobacco significantly decreased following drought treatment for both varieties (**Figure 1E**). In comparison to the control group (CK), Q96 exhibited reductions of 14.9%, 26.5%, and 38.9% during the D1, D2, and D3 stages, respectively, while Y116 exhibited decreases of 25.9%, 33.7%, and 52.0%. Following the application of SLs, the dry weight per plant in the T1, T2, and T3 stages increased compared to D1, D2, and D3, with Q96 increasing by 14.8%, 27.9%, and 29.9%, respectively, and Y116 increasing by 32.5%, 23.3%, and 59.0%.

### 3.2 Effects of exogenous SLs on chlorophyll content in leaves of flue-cured tobacco seedlings under different degrees of drought stress

#### 3.2.1 Chlorophyll a content

**Figure 2:**
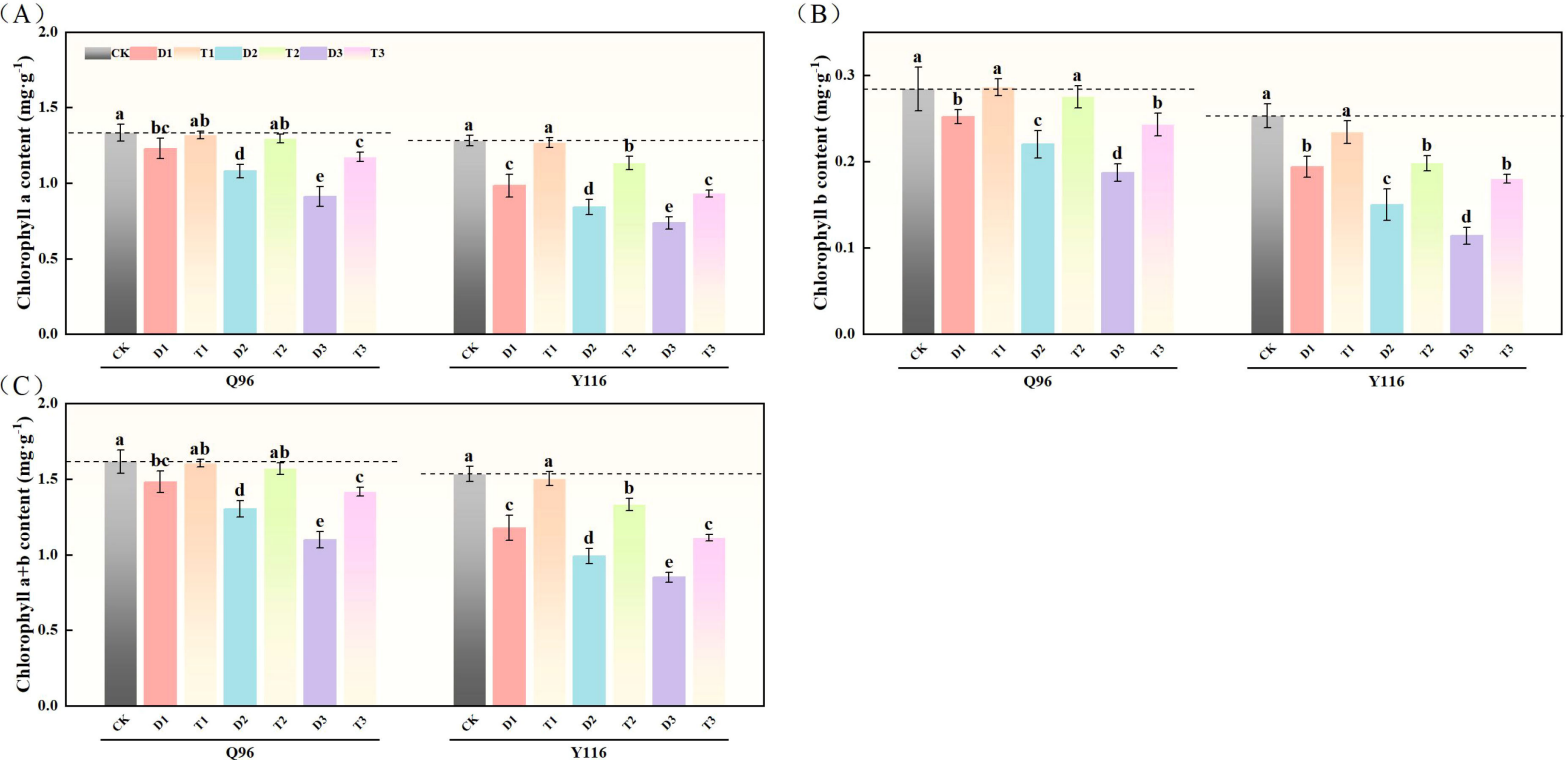
Effect of exogenous SLs on chlorophyll content of flue-cured tobacco seedlings under different drought stress. **(A)** Effects of different drought stress on chlorophyll a content of flue-cured tobacco seedlings, **(B)** Effects of different drought stress on chlorophyll b content of flue-cured tobacco seedlings, **(C)** Effects of different drought stress on chlorophyll a and b content of flue-cured tobacco seedling. The error bars represent the standard error for each group, and different letters (a, b, c, d, e) indicate significant differences (p< 0.05). “Q96” and “Y116” mean flue-cured tobacco varieties Qinyan 96 and Yunyan 116, respectively. CK, D1, D2, D3, T1, T2,and T3 represent adequate water supply (75–80% soil water holding capacity), light drought (55–60% soil water holding capacity), moderate stress (45–50% soil water holding capacity) and severe drought (35–40% soil water holding capacity), light drought (55–60% soil water holding capacity) + 0.2 mg.L^-1^SLs, moderate stress (45–50% soil water holding capacity) + 0.2 mg.L^-1^SLs and severe drought (35–40% soil water holding capacity) + 0.2 mg.L^-1^SLs.

As drought stress increased, the chlorophyll a content of Q96 and Y116 gradually decreased. **Figure 2A** shows that the chlorophyll a content under D1, D2, and D3 treatments significantly decreased compared to the control group (CK), with Q96 decreasing by 7.7%, 18.9%, and 31.5%, respectively, and Y116 decreasing by 23.3%, 34.3%, and 42.6%. Following the application of SLs treatment, no significant change in chlorophyll a content was observed for T1 compared to CK. When comparing T1, T2, and T3 with D1, D2, and D3, chlorophyll a content significantly increased under SLs application at the same drought levels, with Q96 increasing by 7.3%, 19.9%, and 28.6%, and Y116 increasing by 29.1%, 34.8%, and 26.6%.

#### 3.2.2 Chlorophyll b content

Following drought treatment, the chlorophyll b content of both tobacco varieties significantly decreased (**Figure 2B**). In comparison to the control group (CK), the chlorophyll b content of Q96 under D1, D2, and D3 treatments decreased by 11.2%, 22.4%, and 34.3%, respectively, while Y116 decreased by 23.2%, 40.7%, and 54.9%. Following the application of SLs treatment, the chlorophyll b content at T1, T2, and T3 stages significantly increased compared to D1, D2, and D3, with Q96 increasing by 13.3%, 24.7%, and 30.0%, and Y116 increasing by 20.6%, 32.2%, and 58.2%.

### 3.3 Effects of exogenous SLs on leaf photosynthetic parameters of flue-cured tobacco seedlings under different degrees of drought stress

#### 3.3.1 Photosynthetic rates

**Figure 3:**
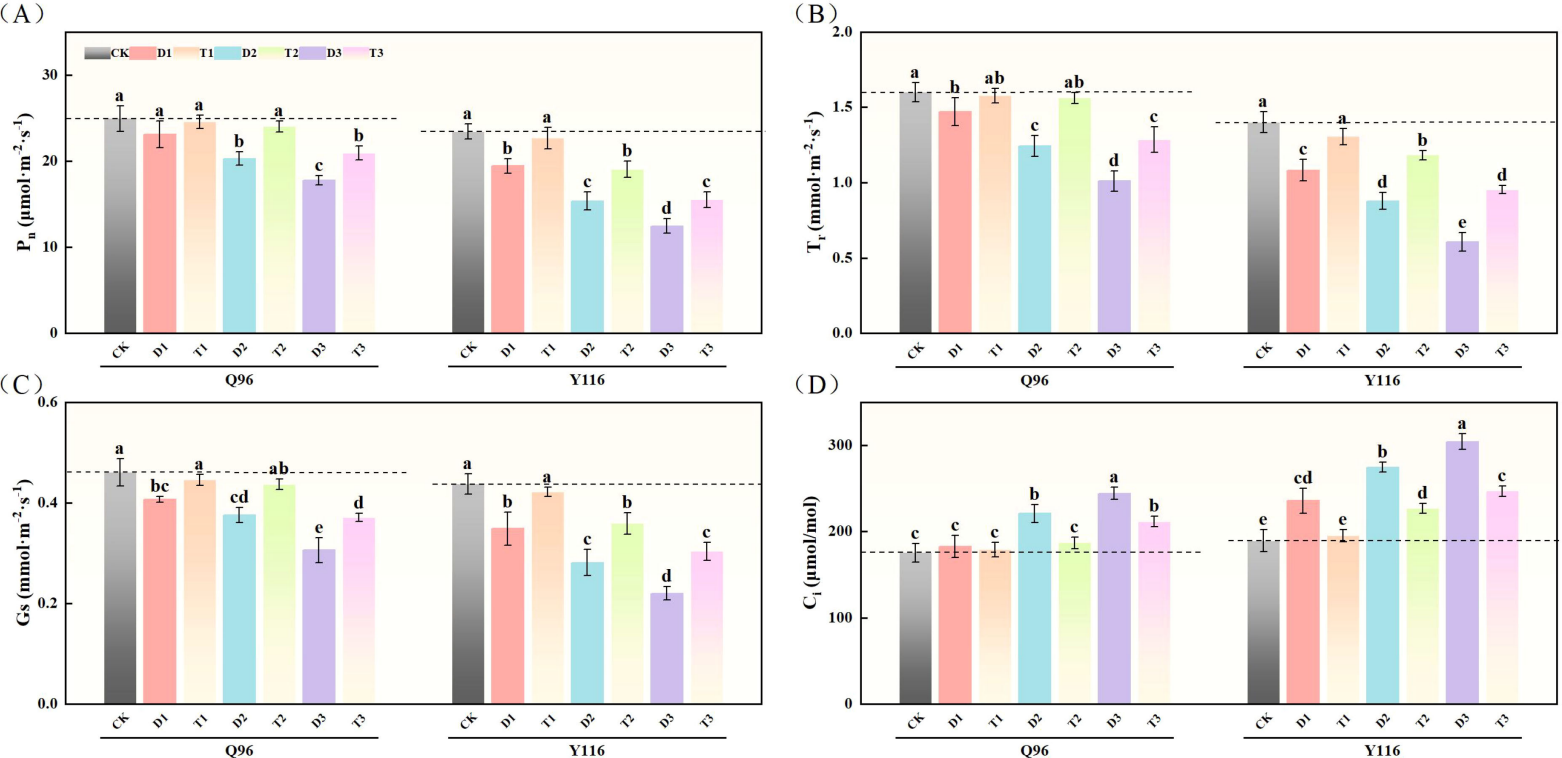
Effects of exogenous SLs on photosynthetic parameters of flue-cured tobacco seedlings under different degrees of drought stress. **(A)** Effects of different drought stress on P_n_ of flue-cured tobacco seedlings, **(B)** Effects of different drought stress on T_r_ of flue-cured tobacco seedlings, **(C)** Effects of different drought stress on G_s_ of flue-cured tobacco seedling. **(D)** Effects of different drought stress on C_i_ of flue-cured tobacco seedlings. The error bars represent the standard error for each group, and different letters (a, b, c, d, e) indicate significant differences (p< 0.05). “Q96” and “Y116” mean flue-cured tobacco varieties Qinyan 96 and Yunyan 116, respectively. CK, D1, D2, D3, T1, T2,and T3 represent adequate water supply (75–80% soil water holding capacity), light drought (55–60% soil water holding capacity), moderate stress (45–50% soil water holding capacity) and severe drought (35–40% soil water holding capacity), light drought (55–60% soil water holding capacity) + 0.2 mg.L^-1^SLs, moderate stress (45–50% soil water holding capacity) + 0.2 mg.L^-1^SLs and severe drought (35–40% soil water holding capacity) + 0.2 mg.L^-1^SLs.

After drought treatment, the P_n_ of both tobacco varieties significantly decreased (**Figure 3A**). The P_n_ of Q96 under D1, D2, and D3 treatments decreased by 7.4%, 18.6%, and 28.7% compared to the control group (CK), while Y116 exhibited decreases of 17.1%, 34.4%, and 46.8%, respectively. Following the application of SLs treatment, the P_n_ in T1, T2, and T3 treatments showed recovery compared to D1, D2, and D3, with Q96 increasing by 6.2%, 18.4%, and 17.7%, and Y116 increasing by 16.7%, 23.9%, and 24.5%.

#### 3.3.2 Transpiration rate

As shown in **Figure 3B**, the T_r_ of both Q96 and Y116 decreased due to drought. Compared to CK, Q96 exhibited a decline of 8.0%, 22.3%, and 36.9% under D1, D2, and D3 treatments, while Y116 experienced by 22.7%, 37.3%, and 56.7%, respectively. Following SLs treatment, the T_r_ in T1, T2, and T3 increased compared to D1, D2, and D3, with Q96 exhibiting increases of 7.1%, 25.5%, and 27.3%, and Y116 showing increases of 20.7%, 34.7%, and 57.0%.

#### 3.3.3 Stomatal conductance

Drought treatment led to a significant reduction in G_s_ for both tobacco varieties (**Figure 3C**). Compared to the control group (CK), the G_s_ of Q96 decreased by 11.9%, 18.5%, and 33.7% under D1, D2, and D3 treatments, while Y116 saw declines of 20.3%, 35.9%, and 49.8%. Following the application of SLs treatment, the G_s_ in T1, T2, and T3 showed improvements compared to D1, D2, and D3, with Q96 increasing by 9.8%, 16.1%, and 21.5%, and Y116 increasing by 20.9%, 27.9%, and 37.8%.

#### 3.3.4 Substomatal cavity CO2 concentration

**Figure 3D** shows that after drought treatment, the C_i_ of Q96 and Y116 significantly increased. Compared to CK, C_i_ in Q96 under D1, D2, and D3 rose by 4.2%, 25.9%, and 39.3%, while Y116 experienced increases of 24.7%, 45.2%, and 60.9%. Under the influence of SLs treatment, the C_i_ in T1, T2, and T3 decreased compared to D1, D2, and D3, with Q96 decreasing by 2.0%, 15.4%, and 13.4%, and Y116 decreasing by 17.1%, 17.3%, and 18.8%.

### 3.4 Effects of exogenous SLs on chlorophyll fluorescence characteristics of flue-cured tobacco seedlings under different degrees of drought stress

#### 3.4.1 Maximal photochemical efficiency (F_v_/F_m_)

**Figure 4:**
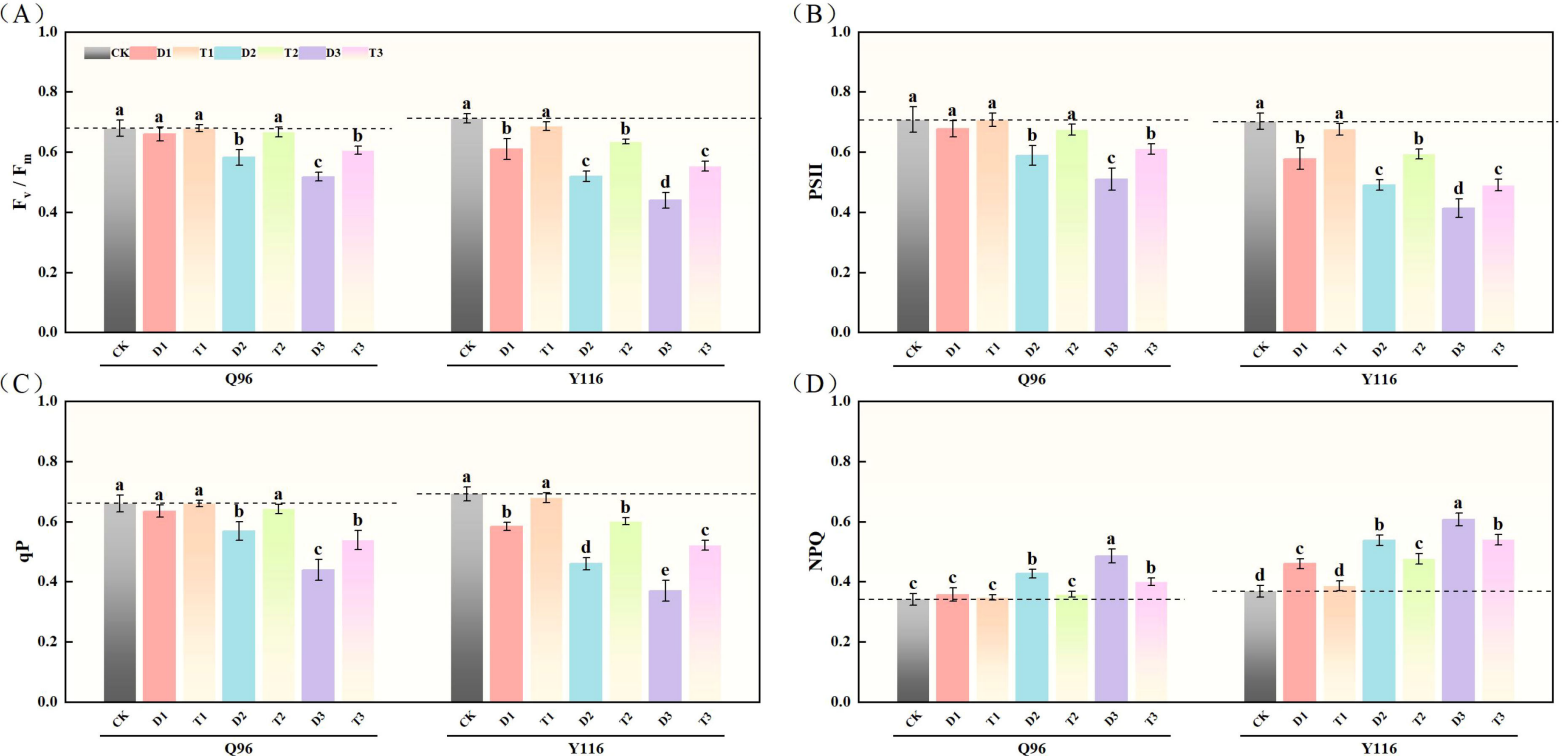
Effects of exogenous SLs on chlorophyll fluorescence parameters of flue-cured tobacco seedlings under different drought stress. **(A)** Effects of different drought stress on F_v_/F_m_ of flue-cured tobacco seedlings, **(B)** Effects of different drought stress on PSII of flue-cured tobacco seedlings, **(C)** Effects of different drought stress on qP of flue-cured tobacco seedling.**(D)** Effects of different drought stress on NPQ of flue-cured tobacco seedlings. The error bars represent the standard error for each group, and different letters (a, b, c, d, e) indicate significant differences (p< 0.05). “Q96” and “Y116” mean flue-cured tobacco varieties Qinyan 96 and Yunyan 116, respectively. CK, D1, D2, D3, T1, T2,and T3 represent adequate water supply (75–80% soil water holding capacity), light drought (55–60% soil water holding capacity), moderate stress (45–50% soil water holding capacity) and severe drought (35–40% soil water holding capacity), light drought (55–60% soil water holding capacity) + 0.2 mg.L^-1^SLs, moderate stress (45–50% soil water holding capacity) + 0.2 mg.L^-1^SLs and severe drought (35–40% soil water holding capacity) + 0.2 mg.L^-1^SLs.

With the worsening drought, the F_v_/F_m_ of Q96 and Y116 showed a gradual decline. **Figure 4A** illustrates that under D1, D2, and D3 treatments, F_v_/F_m_ significantly decreased compared to the control group (CK), with Q96 decreasing by 2.8%, 14.3%, and 23.7%, while Y116 decreased by 14.2%, 27.0%, and 38.2%, respectively. Following the application of SLs treatment, there was no significant change in F_v_/F_m_ between T1 and CK. Comparing T1, T2, and T3 with D1, D2, and D3 reveals that after spraying SLs, F_v_/F_m_ significantly improved under the same drought conditions, with Q96 increasing by 3.0%, 14.7%, and 17.1%, and Y116 increasing by 12.3%, 22.3%, and 25.8%.

#### 3.4.2 Photosystem II

After drought treatment, the PSII of both tobacco varieties significantly decreased (**Figure 4B**). Compared to the control group (CK), the PSII of Q96 declined by 4.4%, 17.0%, and 28.0% under D1, D2, and D3 treatments, while Y116 declined by 17.7%, 30.2%, and 41.2%, respectively. Following the application of SLs treatment, the PSII of T1, T2, and T3 increased compared to D1, D2, and D3, with Q96 increasing by 4.4%, 14.9%, and 19.6%, and Y116 increasing by 16.9%, 20.9%, and 18.7%.

#### 3.4.3 Photochemical quenching coefficient

In **Figure 4C**, we can see that qP of Q96 and Y116 decreased under drought stress. Under D1, D2, and D3 treatments, Q96 decreased by 4.0%, 13.9%, and 33.6% compared to CK, while Y116 decreased by 15.6%, 33.5%, and 46.6%. Following SLs treatment, the qP of T1, T2, and T3 increased compared to D1, D2, and D3, with Q96 increasing by 4.0%, 12.8%, and 22.5%, and Y116 increasing by 16.3%, 30.8%, and 40.9%.

#### 3.4.4 Non-photochemical quenching

The results in **Figure 4D** show that NPQ of Q96 and Y116 increased under drought conditions. Under D1, D2, and D3 treatments, NPQ of Q96 increased by 4.5%, 25.0%, and 42.1% compared to CK, while Y116’s increase was 25.0%, 46.3%, and 65.0%. Following SLs treatment, NPQ of T1, T2, and T3 decreased compared to D1, D2, and D3, with Q96 decreasing by 2.6%, 16.1%, and 17.5%, and Y116 decreasing by 16.0%, 11.6%, and 11.0%.

### 3.5 Effects of exogenous SLs on superoxide anion production rate of flue-cured tobacco seedlings under different degrees of drought stress

**Figure 5:**
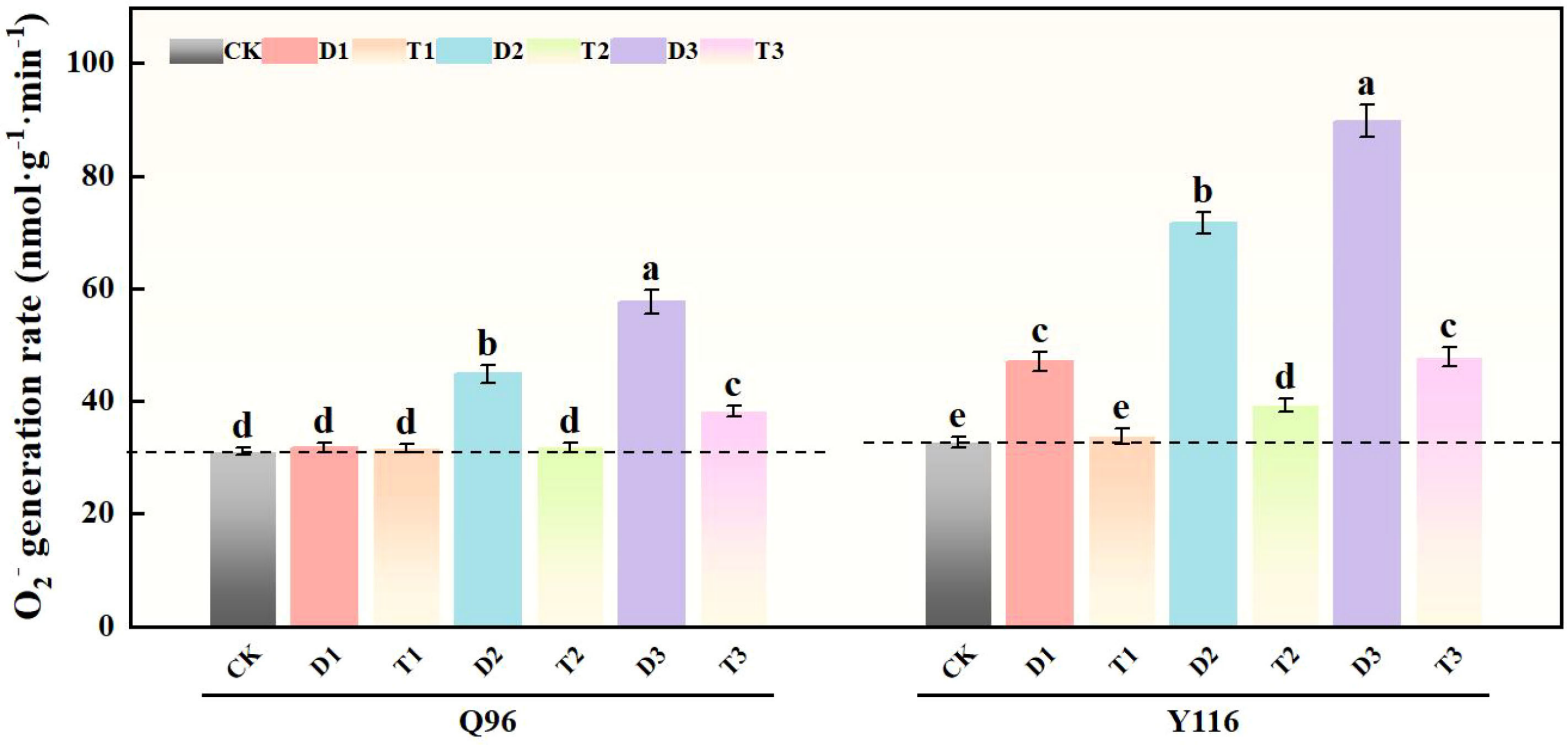
Effects of exogenous SLs on O_2_.^-^ in flue-cured tobacco seedlings under different drought stress. The error bars represent the standard error for each group, and different letters (a, b, c, d, e) indicate significant differences (p< 0.05). “Q96” and “Y116” mean flue-cured tobacco varieties Qinyan 96 and Yunyan 116, respectively. CK, D1, D2, D3, T1, T2,and T3 represent adequate water supply (75–80% soil water holding capacity), light drought (55–60% soil water holding capacity), moderate stress (45–50% soil water holding capacity) and severe drought (35–40% soil water holding capacity), light drought (55–60% soil water holding capacity) + 0.2 mg.L^-1^SLs, moderate stress (45–50% soil water holding capacity) + 0.2 mg.L^-1^SLs and severe drought (35–40% soil water holding capacity) + 0.2 mg.L^-1^SLs.

Under drought stress, the generation rate of superoxide anion (O_2_^-^) in Q96 and Y116 exhibited an upward trend, with Y116 being more pronounced than Q96. According to the data in **Figure 5**, treatments D1, D2, and D3 significantly increased the O_2_^-^ generation rate compared to CK, with Q96 increasing by 2.3%, 44.4%, and 85.5%, respectively; while Y116 increased by 44.1%, 119.7%, and 174.9%. At the same drought level of drought, the application of SLs in T1, T2, and T3 compared to D1, D2, and D3 resulted in a significant increase in Q96’s O_2_^-^ generation rate of 0.2%, 29.0%, and 33.6%, whereas Y116 increased by 28.0%, 45.2%, and 46.7%.

### 3.6 Effects of exogenous SLs on MDA content of flue-cured tobacco seedlings under different degrees of drought stress

**Figure 6:**
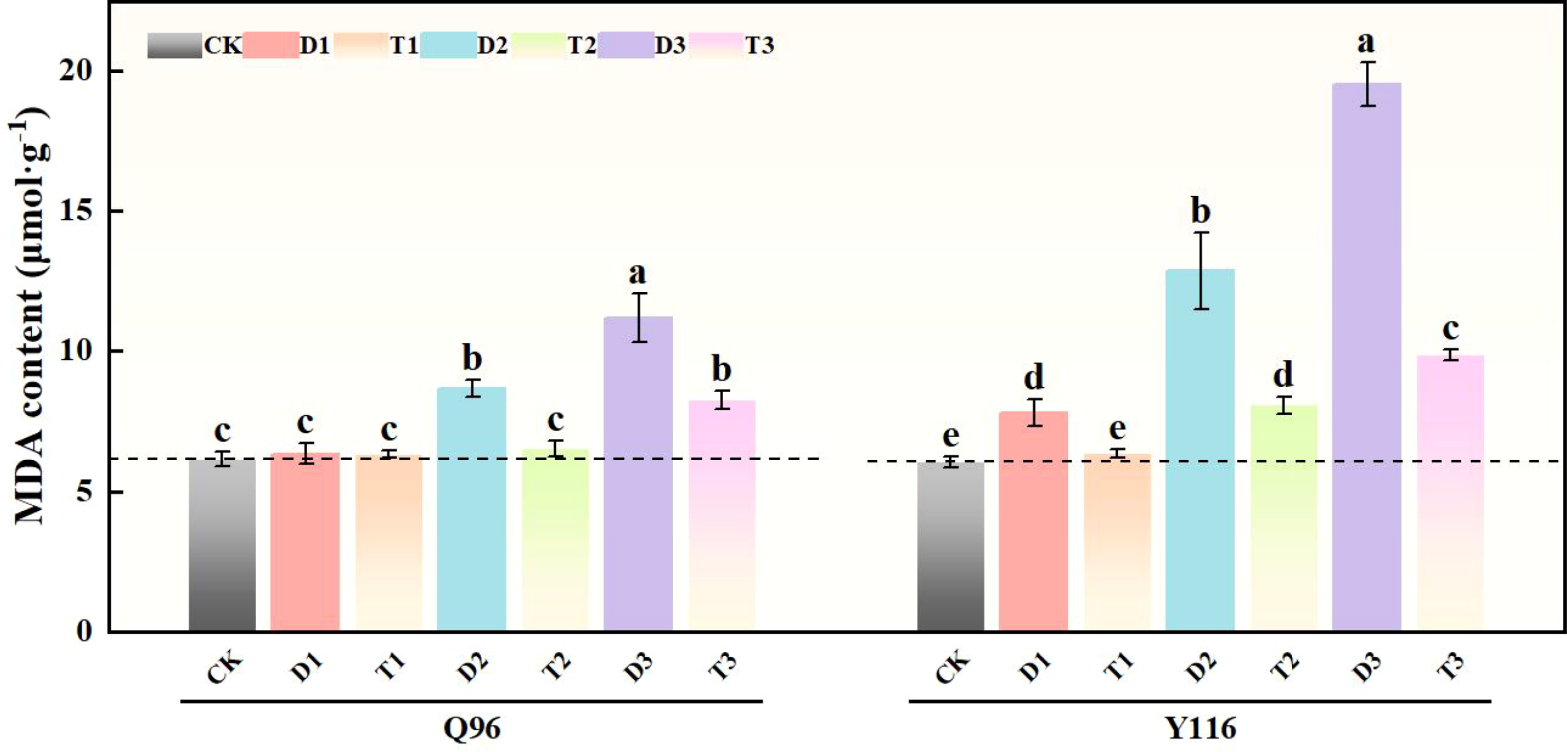
Effect of exogenous SLs on MDA content of flue-cured tobacco seedlings under different drought stress. The error bars represent the standard error for each group, and different letters (a, b, c, d, e) indicate significant differences (p< 0.05). “Q96” and “Y116” mean flue-cured tobacco varieties Qinyan 96 and Yunyan 116, respectively. CK, D1, D2, D3, T1, T2,and T3 represent adequate water supply (75–80% soil water holding capacity), light drought (55–60% soil water holding capacity), moderate stress (45–50% soil water holding capacity) and severe drought (35–40% soil water holding capacity), light drought (55–60% soil water holding capacity) + 0.2 mg.L^-1^SLs, moderate stress (45–50% soil water holding capacity) + 0.2 mg.L^-1^SLs and severe drought (35–40% soil water holding capacity) + 0.2 mg.L^-1^SLs.

Under drought conditions, the generation rate of malondialdehyde (MDA) content in Q96 and Y116 increased, with Y116 exhibiting a more significant rise (**Figure 6**). The drought treatment groups D1, D2, and D3 resulted in a marked increase in MDA content compared to CK, with Q96 increasing by 3.1%, 41.1%, and 81.9%, respectively, while Y116 exhibited increases of 29.3%, 112.9%, and 222.8%. In comparison to D1, D2, and D3, the application of SLs treatment in T1, T2, and T3 resulted in an increase in Q96’s MDA content of 0.4%, 24.8%, and 26.2%, whereas Y116 exhibited a larger increase of 18.9%, 37.3%, and 49.4%.

### 3.7 Effects of exogenous SLs on antioxidant oxidase activity of flue-cured tobacco seedlings under different degrees of drought stress

#### 3.7.1 Superoxide dismutase

**Figure 7:**
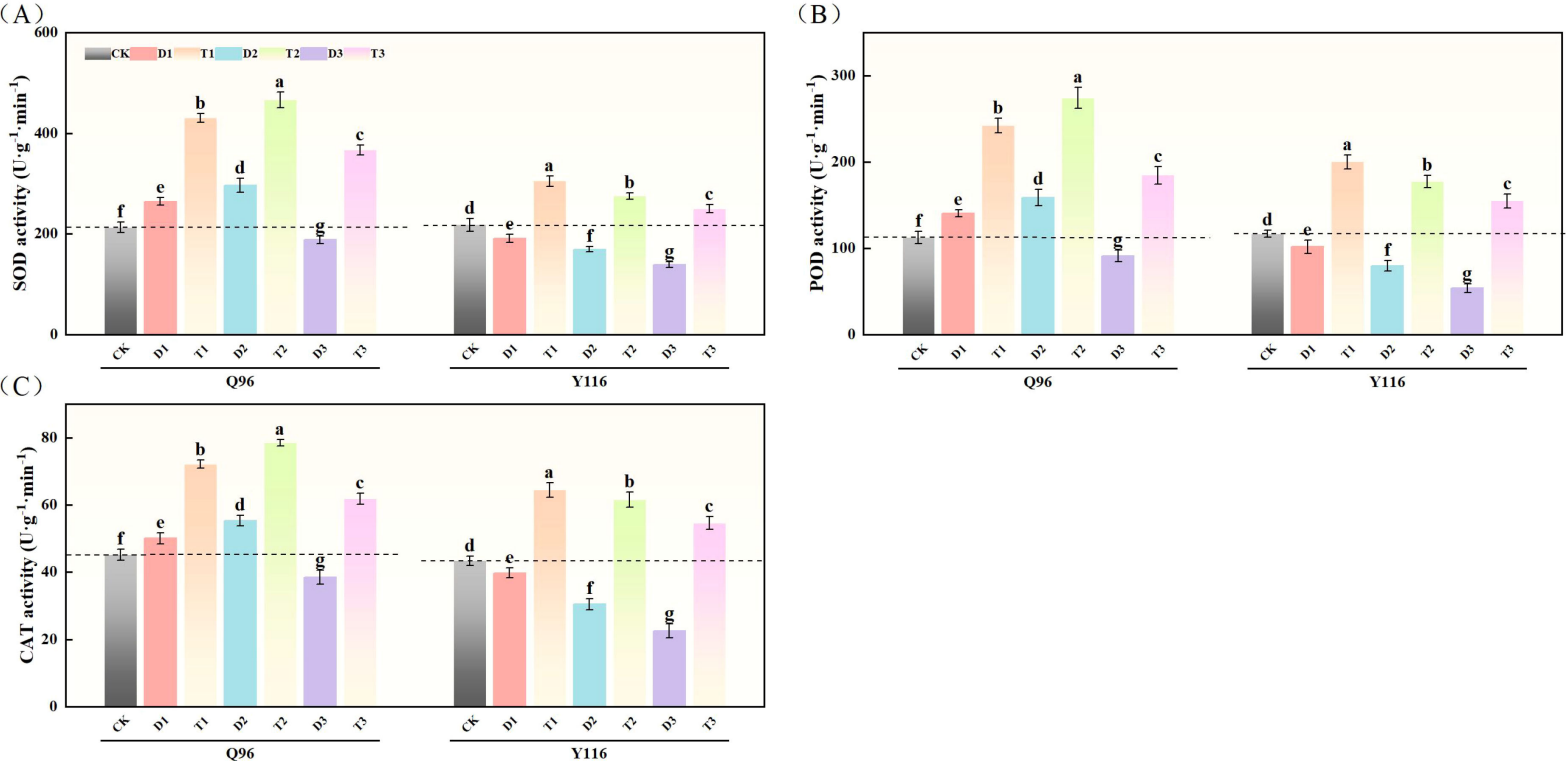
Effects of exogenous SLs on antioxidant enzyme activities of flue-cured tobacco seedlings under different drought stress. **(A)** Effects of different drought stress on SOD activity of flue-cured tobacco seedlings, **(B)** Effects of different drought stress on POD activity of flue-cured tobacco seedlings, **(C)** Effects of different drought stress on CAT activity of flue-cured tobacco seedling. The error bars represent the standard error for each group, and different letters (a, b, c, d, e) indicate significant differences (p< 0.05). “Q96” and “Y116” mean flue-cured tobacco varieties Qinyan 96 and Yunyan 116, respectively. CK, D1, D2, D3, T1, T2,and T3 represent adequate water supply (75–80% soil water holding capacity), light drought (55–60% soil water holding capacity), moderate stress (45–50% soil water holding capacity) and severe drought (35–40% soil water holding capacity), light drought (55–60% soil water holding capacity) + 0.2 mg.L^-1^SLs, moderate stress (45–50% soil water holding capacity) + 0.2 mg.L^-1^SLs and severe drought (35–40% soil water holding capacity) + 0.2 mg.L^-1^SLs.

**Figure 7A** illustrates that compared to CK, the SOD activity of Q96 tobacco increased by 24.2% and 39.2% under D1 and D2 treatments, respectively, but decreased by 11.4% under D3 treatment compared to CK. In contrast, the SOD activity of Y116 decreased by 12.3%, 22.1%, and 36.2% under D1, D2, and D3 treatments, respectively. Following the application of SLs treatment in T1, T2, and T3, the SOD activity significantly increased compared to D1, D2, and D3, with Q96 exhibiting increases of 62.6%, 57.3%, and 94.5%, while Y116 increased by 59.5%, 62.0%, and 79.9%.

#### 3.7.2 Peroxidase

According to **Figure 7B**, the POD activity of Q96 tobacco increased by 24.9% and 41.0% compared to CK under D1 and D2 treatments, respectively, but decreased by 18.8% under D3 treatment. The POD activity of Y116 decreased by 12.8%, 31.8%, and 53.8% under the same drought treatments. Following the application of SLs treatment in T1, T2, and T3, the POD activity of Q96 significantly increased compared to D1, D2, and D3, with increases of 72.5%, 72.8%, and 101.9%, while Y116 exhibited increases of 96.7%, 122.5%, and 187.1%.

#### 3.7.3 Catalase

**Figure 7C** indicates that the CAT activity of Q96 tobacco increased by 10.9% and 22.5% compared to CK under D1 and D2 treatments, respectively, but decreased by 14.7% under D3 treatment. In comparison, the CAT activity of Y116 decreased by 8.4%, 29.9%, and 48.1% under D1, D2, and D3 treatments, respectively. Following the application of SLs treatment, the CAT activity of Q96 showed significant increases compared to D1, D2, and D3, with growth rates of 44.1%, 42.0%, and 60.4%, while Y116 increased by 62.3%, 102.3%, and 142.4%.

### 3.8 Effects of exogenous SLs on the content of osmoregulatory substances in flue-cured tobacco seedlings under different degrees of drought stress

#### 3.8.1 Proline

**Figure 8:**
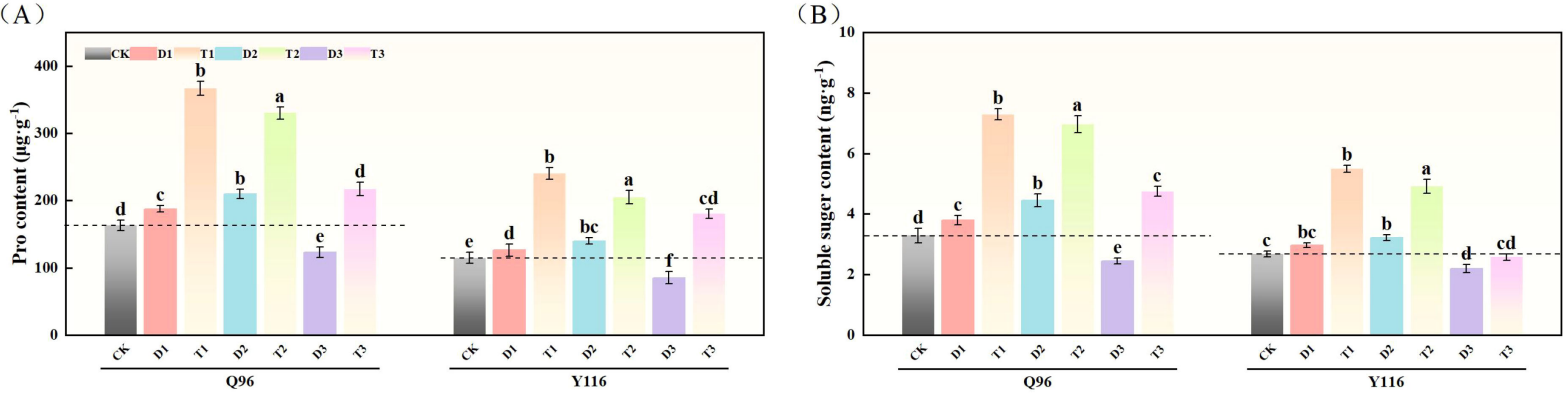
Effect of exogenous SLs on osmotic adjustment substance content of flue-cured tobacco seedlings under different drought stress. **(A)** Effects of different drought stress on Pro content of flue-cured tobacco seedlings, **(B)** Effects of different drought stress on soluble sugar content of flue-cured tobacco seedlings. The error bars represent the standard error for each group, and different letters (a, b, c, d, e) indicate significant differences (p< 0.05). “Q96” and “Y116” mean flue-cured tobacco varieties Qinyan 96 and Yunyan 116, respectively. CK, D1, D2, D3, T1, T2,and T3 represent adequate water supply (75–80% soil water holding capacity), light drought (55–60% soil water holding capacity), moderate stress (45–50% soil water holding capacity) and severe drought (35–40% soil water holding capacity), light drought (55–60% soil water holding capacity) + 0.2 mg.L^-1^SLs, moderate stress (45–50% soil water holding capacity) + 0.2 mg.L^-1^SLs and severe drought (35–40% soil water holding capacity) + 0.2 mg.L^-1^SLs.

As shown in **Figure 8A**, compared to CK, the Pro content in Q96 increased by 15.1% and 28.7% under D1 and D2 treatments, respectively, while Y116 increased by 10.2% and 22.0%. However, under D3 treatment, Q96 decreased by 24.6%, and Y116 decreased by 25.8%. Following the application of SLs treatment in T1, T2, and T3, the Pro content in Q96 exhibited significant increases compared to D1, D2, and D3, with growth rates of 95.7%, 57.4%, and 76.4%, respectively; Y116 increased by 90.1%, 46.5%, and 112.3%.

#### 3.8.2 Soluble sugar content

In **Figure 8B**, illustrates that under D1 and D2 treatments, compared to CK, the soluble sugar content in Q96 increased by 15.6% and 35.5%, respectively, while Y116 increased by 11.1% and 20.6%. However, under D3 treatment, Q96 decreased by 25.6%, and Y116 decreased by 17.9%. Following the application of SLs treatment, the soluble sugar content in Q96 significantly improved when comparing T1, T2, and T3 with D1, D2, and D3, with increases of 92.1%, 56.4%, and 94.3%, respectively; Y116 increased by 85.0%, 52.5%, and 17.2%.

### 3.9 Effects of exogenous SLs on carbon and nitrogen metabolic enzyme activities of flue-cured tobacco seedlings under different degrees of drought stress

#### 3.9.1 Nitrate reductase

**Figure 9:**
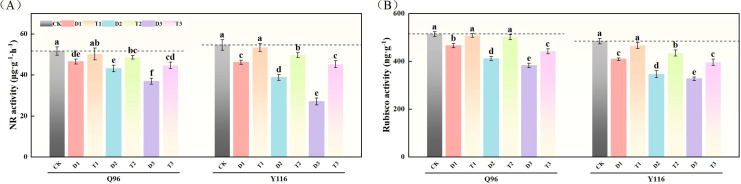
Effects of exogenous SLs on activities of carbon and nitrogen metabolism enzymes in flue-cured tobacco seedlings under different degrees of drought stress. **(A)** Effects of different drought stress on NR activity of flue-cured tobacco seedlings, **(B)** Effects of different drought stress on Rubisco activity of flue-cured tobacco seedlings. The error bars represent the standard error for each group, and different letters (a, b, c, d, e) indicate significant differences (p< 0.05). “Q96” and “Y116” mean flue-cured tobacco varieties Qinyan 96 and Yunyan 116, respectively. CK, D1, D2, D3, T1, T2,and T3 represent adequate water supply (75–80% soil water holding capacity), light drought (55–60% soil water holding capacity), moderate stress (45–50% soil water holding capacity) and severe drought (35–40% soil water holding capacity), light drought (55–60% soil water holding capacity) + 0.2 mg.L^-1^SLs, moderate stress (45–50% soil water holding capacity) + 0.2 mg.L^-1^SLs and severe drought (35–40% soil water holding capacity) + 0.2 mg.L^-1^SLs.

With the increasing drought stress, the NR activity of Q96 and Y116 gradually decreased. As shown in **Figure 9A**, the NR activity under D1, D2, and D3 treatments significantly decreased compared to CK, with Q96 declining by 9.9%, 16.5%, and 28.5%, respectively, while Y116 decreased by 15.3%, 28.9%, and 50.5%. Following the application of SLs, there was no significant change in NR activity for T1 compared to CK. When comparing T1, T2, and T3 with D1, D2, and D3, the NR activity significantly increased following SLs application application under the same drought conditions, with Q96 exhibiting increases of 7.8%, 12.6%, and 21.1%, respectively, and Y116 increasing by 15.4%, 28.1%, and 67.1%.

#### 3.9.2 Ribulose 1,5-diphosphate carboxylase (Rubisco)

Following drought treatment, the Rubisco activity in both tobacco varieties significantly decreased (**Figure 9B**). For Q96, the Rubisco activity under D1, D2, and D3 treatments was significantly reduced compared to the control group (CK) by 9.3%, 19.9%, and 25.6%, respectively. Y116 similarly exhibited decreases of 15.6%, 28.6%, and 32.5%. Following the application of SLs, the Rubisco activity in T1, T2, and T3 exhibited recovery compared to D1, D2, and D3, with Q96 increasing by 9.0%, 21.7%, and 15.3%, respectively, and Y116 increasing by 13.9%, 25.7%, and 21.0%.

### 3.10 Effects of exogenous SLs on endogenous hormone content of tobacco seedlings under different degrees of drought stress

#### 3.10.1 Auxin (IAA)

**Figure 10:**
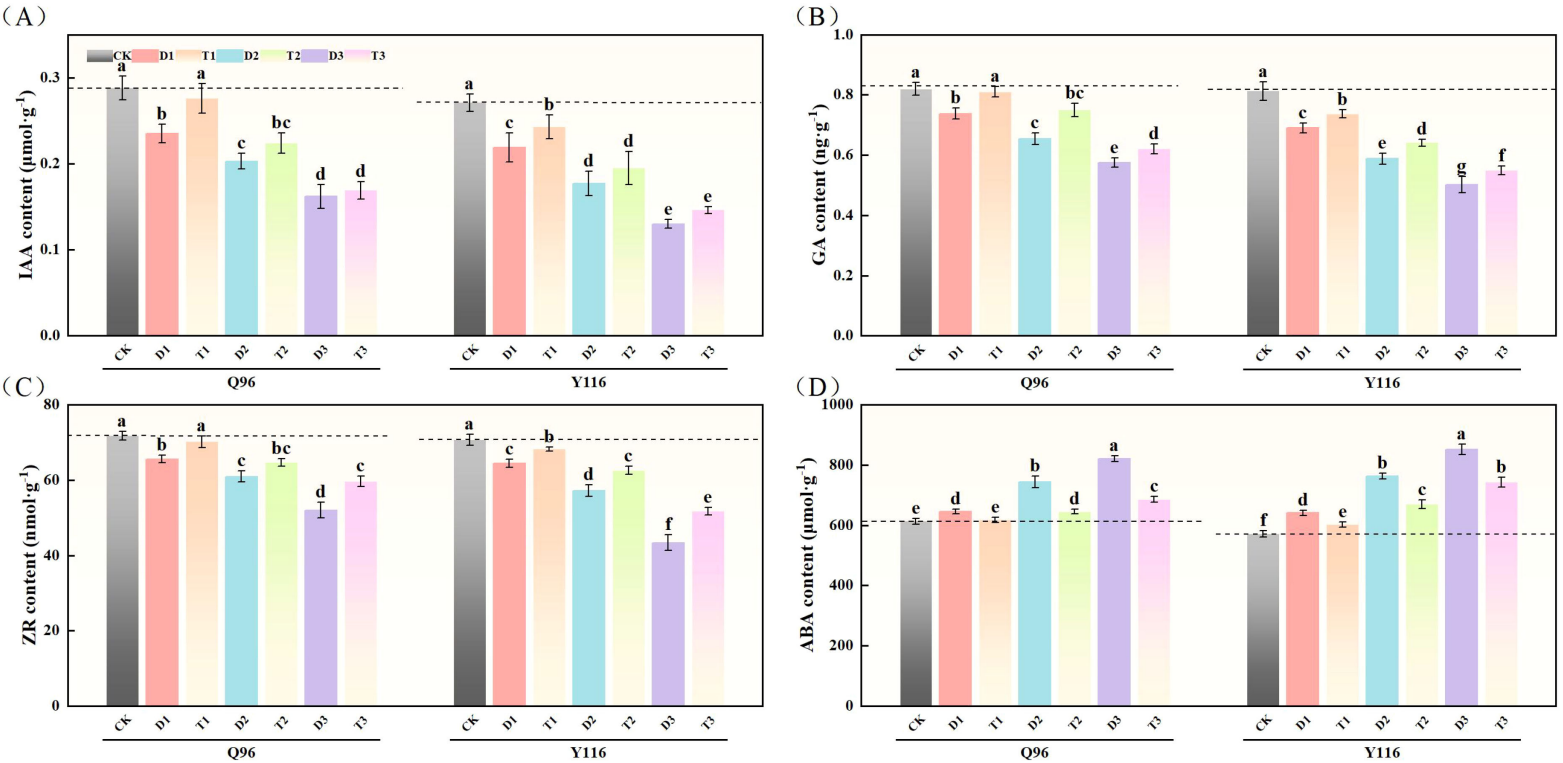
Effect of exogenous SLs on endogenous hormone content of flue-cured tobacco seedlings under. **(A)** Effects of different drought stress on IAA content of flue-cured tobacco seedlings, **(B)** Effects of different drought stress on GA content length of flue-cured tobacco seedlings, **(C)** Effects of different drought stress on ZR content width of flue-cured tobacco seedling.**(D)** Effects of different drought stress on ABA content area of flue-cured tobacco seedlings. The error bars represent the standard error for each group, and different letters (a, b, c, d, e) indicate significant differences (p< 0.05). “Q96” and “Y116” mean flue-cured tobacco varieties Qinyan 96 and Yunyan 116, respectively. CK, D1, D2, D3, T1, T2,and T3 represent adequate water supply (75–80% soil water holding capacity), light drought (55–60% soil water holding capacity), moderate stress (45–50% soil water holding capacity) and severe drought (35–40% soil water holding capacity), light drought (55–60% soil water holding capacity) + 0.2 mg.L^-1^SLs, moderate stress (45–50% soil water holding capacity) + 0.2 mg.L^-1^SLs and severe drought (35–40% soil water holding capacity) + 0.2 mg.L^-1^SLs.

With the worsening drought, the IAA levels of Q96 and Y116 showed a gradual decline. **Figure 10A** illustrates that under D1, D2, and D3 treatments, the IAA significantly decreased compared to CK, with Q96 showing reductions of 18.3%, 29.4%, and 43.7%, while Y116 decreased by 19.0%, 34.6%, and 52.0%, respectively. Following the application of SLs, there was no significant change in IAA between T1 and CK. A comparison of T1, T2, T3 with D1, D2, D3 revealed that following the application of SLs, IAA significantly increased under the same drought conditions, with Q96 increasing by 17.6%, 10.2%, and 4.5%, and Y116 increasing by 10.8%, 10.2%, and 12.1%, respectively.

#### 3.10.2 Gibberellic acid

In **Figure 10B**, both Q96 and Y116 exhibited a decline in GA due to drought. Compared to CK, Q96 experienced decreases of 10.0%, 20.3%, and 29.9% under D1, D2, and D3 treatments, while Y116 experienced declines of 15.1%, 27.6%, and 38.2%. Following the application of SLs, GA levels in T1, T2, and T3 were higher than those in D1, D2, and D3, with Q96 increasing by 9.7%, 14.5%, and 8.0%, and Y116 increasing by 6.8%, 9.0%, and 9.4%.

#### 3.10.3 Ribosylzeatin

Following drought treatment, ZR levels in both tobacco varieties significantly declined (**Figure 10C**). Under D1, D2, and D3 treatments, the ZR levels in Q96 significantly decreased by 8.6%, 15.1%, and 27.6% compared to the control group (CK), while Y116 experienced declines of 8.8%, 19.0%, and 38.7%. After the application of SLs, ZR levels in T1, T2, and T3 exhibited recovery compared to D1, D2, and D3, with Q96 increasing by 7.1%, 6.0%, and 14.8%, and Y116 increasing by 5.8%, 9.4%, and 19.2%.

#### 3.10.4 Abscisic acid

The results in **Figure 10D** show that ABA levels in Q96 and Y116 increased under drought conditions. Under D1, D2, and D3 treatments, the ABA levels in Q96 rose by 5.3%, 21.4%, and 34.0% compared to the control group (CK), while Y116 experienced increases of 12.2%, 33.6%, and 49.1%, respectively. Following the application of SLs, ABA levels in T1, T2, and T3 decreased compared to D1, D2, and D3, with Q96 declining by 4.3%, 13.3%, and 16.6%, and Y116 declining by 6.0%, 12.2%, and 12.8%.

### 3.11 Cluster analysis and correlation analysis of 29 traits

**Figure 11:**
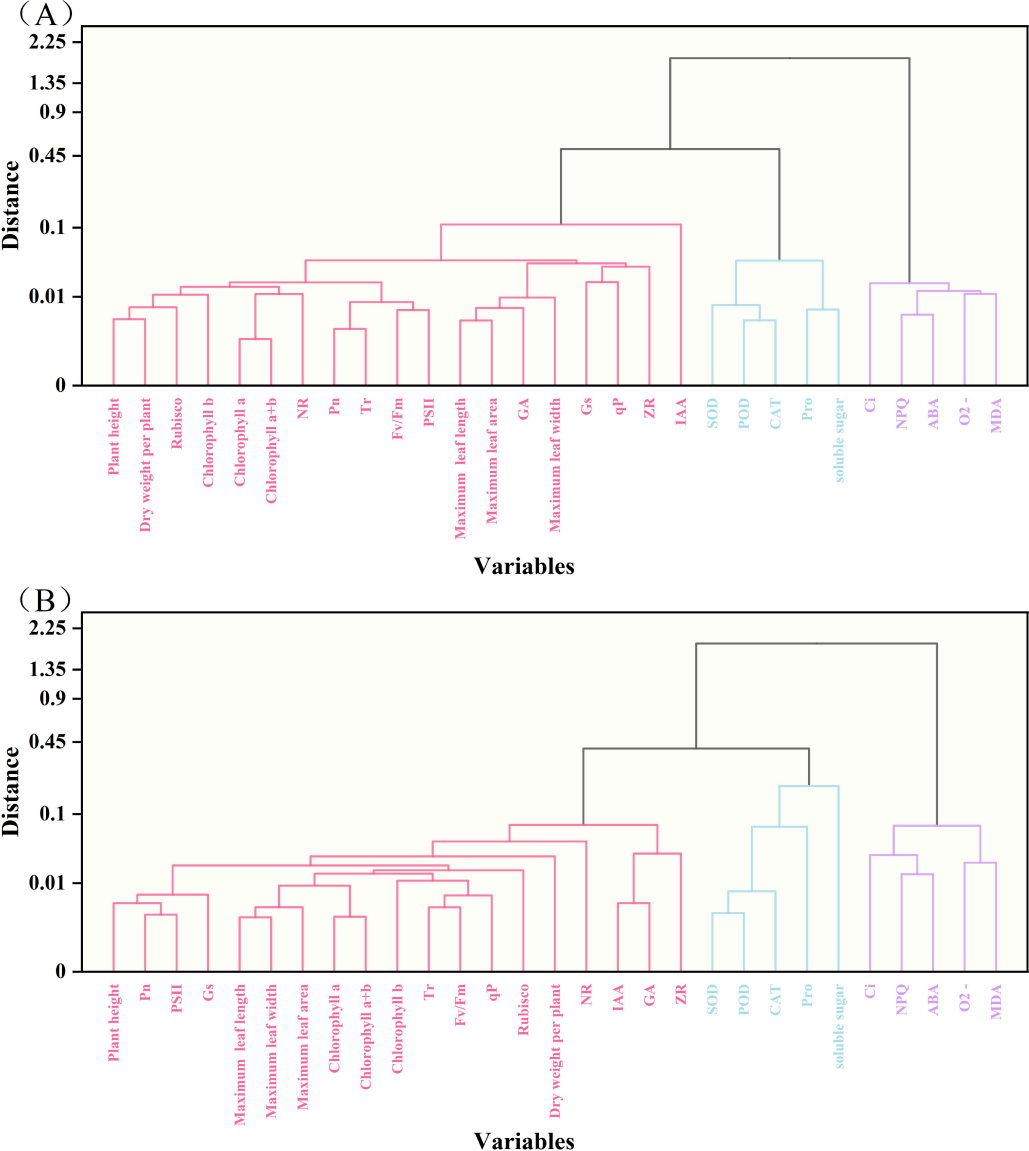
Cluster analysis of drought tolerance traits in two tobacco varieties. **(A)** Cluster analysis of drought tolerance traits in Q96, and **(B)** Cluster analysis of drought tolerance traits in Y116. The three colors from left to right represent Cluster 1, Cluster 2 and Cluster 3, respectively.

**Figure 12:**
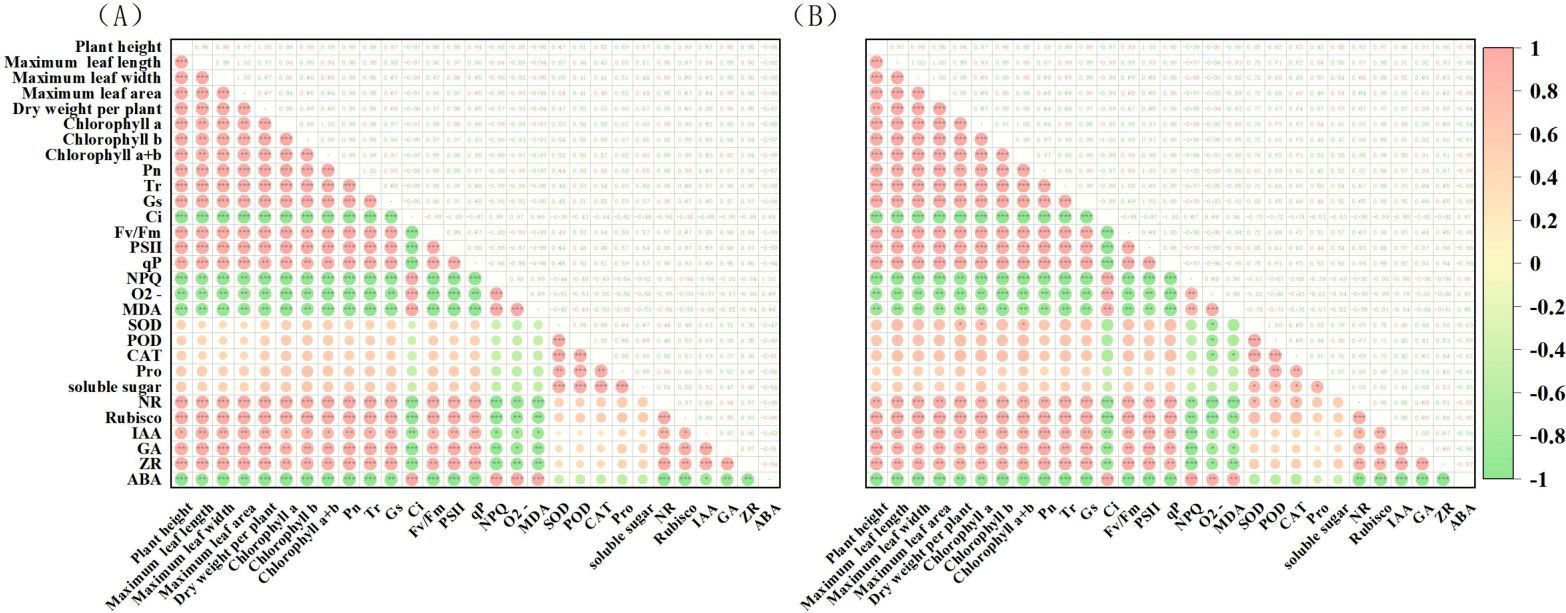
Correlation analysis of drought tolerance traits in two tobacco varieties **(A)** Correlation analysis of drought tolerance traits in Q96, and **(B)** Correlation analysis of drought tolerance traits in Y116. *, **, and *** are significantly different at p ≤ 0.05, 0.01, and 0.001 significant levels, respectively.

The potential drought tolerance traits of the two tobacco varieties (29 traits in total) were analyzed through clustering. The traits of the Q96 variety (**Figure 11A**) were grouped into three clusters: Cluster 1 containing 19 traits, Cluster 2 containing 5 traits, and Cluster 3 containing 5 traits. The clustering analysis for the Y116 variety (**Figure 11B**) yielded the same result as for the Q96 variety, with traits grouped into three clusters, and the composition of traits in each cluster being identical. The traits were similarly divided into three clusters, with each cluster containing the same set of traits. Cluster analysis effectively grouped highly correlated traits into the same clusters, suggesting that traits within the same cluster share similar drought resistance. Pearson’s correlation analysis revealed that the drought tolerance traits of the Q96 and Y116 tobacco varieties were highly correlated (**Figure 12**). Highly significant positive correlations were observed among 19 traits, including plant height, maximum leaf length, and maximum leaf width. Positive correlations were also found between Ci, NPQ, O_2_^−^ generation rate, MDA content, and ABA content. These traits showed significant negative correlations with the aforementioned 19 traits. Combining the clustering (**Figure 11**) and correlation analysis plots (**Figure 12**) showed that traits within the same cluster exhibited significant correlations, it was further demonstrated that these traits are closely interrelated and may collectively contribute to the drought response mechanisms in tobacco.

### 3.12 Principal component analysis of different varieties of tobacco

**Table 2** presents the identification of two principal components through principal component analysis (PCA) of 29 physiological indices from two tobacco varieties, based on eigenvalues ≥ 1.00. The variance contributions of the two principal components for Q96 were 84.82% and 12.28%, respectively, yielding a cumulative variance contribution of 97.10%. Consequently, the original 29 indices were transformed into two new composite indices. The first principal component exhibited high loadings for factors including plant height, chlorophyll a content, chlorophyll b content, T_r_, F_v_/F_m_, and PSII, which are primarily associated with tobacco agronomic traits, chlorophyll content, and photosynthesis. Conversely, the second principal component encompassed SOD activity, POD activity, CAT activity, proline, and soluble sugar content, which are mainly associated with antioxidant enzyme systems and osmotic adjustment. The variance contributions of the two principal components for Y116 were 86.79% and 9.78%, respectively, resulting in a cumulative variance contribution of 96.57%. Additionally, the 29 indicators were transformed into two composite indices.

**Table 2:** Principal component matrix and cumulative contribution of different tobacco varieties.

Indexes	Variety	Q96		Y116	
	Principal component	1	2	1	2
Eigenvalue		24.60	3.56	25.17	2.84
CR(%)		84.82	12.28	86.79	9.78
CCR(%)		84.82	97.10	86.79	96.57
W_j_		0.87	0.13	0.90	0.10
Eigenvector	Plant height	0.99	0.06	0.98	0.15
	Maximum leaf length	0.97	0.15	1.00	0.03
	Maximum leaf width	0.98	0.19	0.99	0.08
	Maximum leaf area	0.97	0.19	0.99	0.08
	Dry weight per plant	0.99	0.06	0.98	-0.01
	Chlorophyll a	0.99	0.01	0.99	0.00
	Chlorophyll b	0.99	-0.01	0.99	0.11
	Chlorophyll a+b	0.99	0.00	0.99	0.02
	P_n_	0.99	0.09	0.98	0.19
	T_r_	1.00	0.05	0.99	0.08
	G_s_	0.98	0.09	0.99	0.11
	C_i_	0.98	0.15	0.99	0.04
	F_v_/F_m_	0.99	0.05	1.00	0.08
	PSII	0.99	0.11	0.98	0.17
	qP	0.97	0.07	1.00	0.05
	NPQ	0.98	0.09	0.97	0.21
	O_2_^-^	0.96	0.01	0.96	-0.12
	MDA	0.97	0.08	0.95	-0.04
	SOD	-0.52	0.85	-0.77	0.63
	POD	-0.57	0.82	-0.74	0.67
	CAT	-0.58	0.81	-0.75	0.64
	Pro	-0.65	0.72	-0.57	0.80
	soluble sugar	-0.63	0.76	-0.60	0.59
	NR	0.98	0.09	0.98	-0.06
	Rubisco	0.98	0.04	0.98	0.04
	IAA	0.87	0.35	0.90	0.39
	GA	0.95	0.21	0.91	0.39
	ZR	0.96	0.23	0.94	0.22
	ABA	0.98	0.07	0.97	0.18

CR, contribution rate; CCR and cumulative contribution rate. CK: Adequate water supply (75–80% soil water holding capacity), D1: Light drought (55–60% soil water holding capacity), D2: Moderate drought (45–50% soil water holding capacity), D3: Severe drought (35–40% soil water holding capacity), T1: Light drought (55–60% soil water holding capacity) + 0.2 mg.L^-1^SLs, T2: Moderate drought (45–50% soil water holding capacity) + 0.2 mg.L^-1^SLs, T3: Severe drought (35–40% soil water holding capacity) + 0.2 mg.L^-1^SLs.

**Figure 13:**
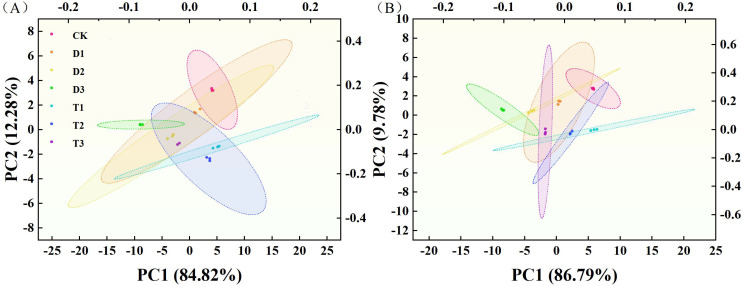
PCA analysis of two tobacco varieties under different treatments. **(A)** PCA analysis of Q96 under different treatments, and **(B)** PCA analysis of Y116 under different treatments. “Q96” and “Y116” mean flue-cured tobacco varieties Qinyan 96 and Yunyan 116, respectively. CK, D1, D2, D3, T1, T2,and T3 represent adequate water supply (75–80% soil water holding capacity), light drought (55–60% soil water holding capacity), moderate stress (45–50% soil water holding capacity) and severe drought (35–40% soil water holding capacity), light drought (55–60% soil water holding capacity) + 0.2 mg.L^-1^SLs, moderate stress (45–50% soil water holding capacity) + 0.2 mg.L^-1^SLs and severe drought (35–40% soil water holding capacity) + 0.2 mg.L^-1^SLs.

The first principal component for Y116 exhibited high loadings for factors such as maximum leaf length, maximum leaf width, T_r_, G_s_, C_i_, F_v_/F_m_, and qP, which are primarily related to tobacco agronomic traits and photosynthesis. The second principal component encompassed SOD activity, POD activity, CAT activity, proline, and soluble sugar content, which are primarily associated with the antioxidant enzyme system and osmotic regulation. PCA results for the Q96 and Y116 tobacco varieties indicated that agronomic traits, photosynthesis, and osmotic regulation significantly influence drought resistance. Furthermore, Q96 exhibited more pronounced differences in response to drought stress under T1 and CK treatments compared to D3 (**Figure 13A**). Y116 also displayed significant differences from D3 under T1, T2, and CK treatments (**Figure 13B**).

### 3.13 Screening and classification of drought tolerance traits in tobacco

Using principal component analysis PCA we identified key traits associated with drought tolerance such as plant height、T_r、_C_i_和SOD activity as the final indicators for drought tolerance assessment However, the data presented in **Table 2** show that the eigenvector values for these key traits are similar to each other. Furthermore, the analyses in **Figures 11** and **12** reveal that these traits exhibit high similarity and strong correlations within Cluster 1, Cluster 2, and Cluster 3. When traits within the same cluster are strongly correlated, changes in one trait can reliably predict changes in the others. Therefore, simplifying some of the redundant indicators in future studies of drought tolerance in tobacco could enhance research efficiency and reduce complexity. The results demonstrated that traits in Cluster 1 were positively correlated with drought tolerance in tobacco, suggesting that these traits are key indicators of enhanced drought resistance. In contrast, traits in Cluster 3 exhibited a negative correlation with drought tolerance, indicating that the increase in these traits may reflect reduced drought resistance in tobacco.

### 3.14 Comprehensive evaluation of different SLs treatments for improving drought tolerance in tobacco

Based on Formula 6, we calculated the affiliation function values for each composite index of the two tobacco varieties across different treatments (**Table 3**) and determined the weights for each composite index (**Table 2**) using Formula 7. The weights for the two composite indices of Q96 were 0.87 and 0.13, while those for Y116 were 0.90 and 0.10. The affiliation function values for each index did not fully represent the level of drought tolerance in tobacco under the various SL treatments. The drought tolerance of the two tobacco varieties under various treatments was ultimately calculated using Formula 8. A higher comprehensive evaluation value was positively correlated with stronger drought tolerance, as illustrated in **Table 3**. Comparison of drought tolerance among different treatments, based on the D value, revealed that Q96 exhibited higher drought tolerance in T1, T2, and CK treatments, whereas Y116 demonstrated higher drought tolerance in T1 and CK treatments.

**Table 3:** Principal component matrix and cumulative contribution of different tobacco varieties.

Variety	Membership function value	Treatment						
		CK	D1	D2	D3	T1	T2	T3
Q96	U(X1)	0.64	0.59	0.38	0.00	1.00	0.96	0.56
	U(X2)	0.42	0.31	0.54	1.00	0.00	0.09	0.29
	D-value	0.62	0.55	0.40	0.13	0.87	0.85	0.53
	Rank	3	4	6	7	1	2	5
Y116	U(X1)	0.85	0.58	0.28	0.00	1.00	0.78	0.56
	U(X2)	0.15	0.26	0.61	1.00	0.00	0.26	0.49
	D-value	0.78	0.55	0.31	0.10	0.90	0.73	0.55
	Rank	2	4	6	7	1	3	4

U, value of the affiliation function. CK: Adequate water supply (75–80% soil water holding capacity), D1: Light drought (55–60% soil water holding capacity), D2: Moderate drought (45–50% soil water holding capacity), D3: Severe drought (35–40% soil water holding capacity), T1: Light drought (55–60% soil water holding capacity) + 0.2 mg.L^-1^SLs, T2: Moderate drought (45–50% soil water holding capacity) + 0.2 mg.L^-1^SLs, T3: Severe drought (35–40% soil water holding capacity) + 0.2 mg.L^-1^SLs.

## 4 Discussion

Drought stress can hinder plant growth and diminish productivity. Under drought conditions, the height, maximum leaf length, maximum leaf width, maximum leaf area, and dry weight per plant of the Q96 and Y116 cultivars were significantly reduced. Our findings indicate that the application of SLs can alleviate this stress, particularly in the water-sensitive Y116 cultivar. Conversely, the drought-tolerant Q96 variety exhibited greater drought tolerance, and the application of SLs further enhanced this tolerance. Xie et al. suggested that SLs enhance crop yield, which aligns with the findings of this study (Xie et al., 2020).

Chloroplasts serve as the primary sites of photosynthesis in plant leaves. Damage to chloroplasts is reflected by changes in chlorophyll content, which indicates the plant’s sensitivity to stress and subsequently affects photosynthetic productivity (Chikkala et al., 2012). We observed that, under drought stress, the chlorophyll content of both tobacco varieties significantly decreased. The reduction in chlorophyll a for Q96 ranged from 7.7% to 31.5%, whereas Y116 experienced an even greater decline. Following the application of 0.2 mg·L⁻¹ SLs, the chlorophyll content of both varieties significantly increased under various drought treatments compared to drought stress alone, indicating that SLs effectively protect chloroplasts in tobacco under drought conditions and enhance chlorophyll production. This finding is consistent with prior research. SLs may upregulate genes associated with chlorophyll synthesis in the light signaling pathway by influencing FHY3 and FAR1, thereby enhancing chlorophyll synthesis in cucumber under stress conditions (Zhang et al., 2022).

Following drought stress, photosynthesis involving CO_2_ in tobacco is inhibited, resulting in the accumulation of CO_2_ in intercellular spaces (Fernández-San Millán et al., 2018). The photosynthetic parameters P_n_, T_r_, and G_s_ of the two tobacco varieties, Q96 and Y116, significantly decreased as drought stress intensified, whereas C_i_ exhibited a significant increase. However, the application of exogenous SLs positively impacted the photosynthetic efficiency of tobacco, with significant enhancements observed in the photosynthetic parameters of both Q96 and Y116. Studies have demonstrated that SL levels can regulate the opening and closing of stomata, thereby reducing water evaporation and enhancing the photosynthetic rate of plants (Kodama et al., 2022; Raja et al., 2023). Therefore, we speculate that SLs may enhance photosynthetic capacity by improving leaf water potential and stomatal conductance.

Monitoring chlorophyll fluorescence parameters provides an accurate reflection of plant photosynthesis and heat dissipation (Murchie and Lawson, 2013). This study monitored the chlorophyll fluorescence parameters of two tobacco varieties subjected to varying degrees of drought stress. Both Q96 and Y116 exhibited significant reductions in F_v_/F_m_, PSII, and qP, whereas NPQ significantly increased. However, the application of SLs generally enhanced these parameters.Given that chlorophyll fluorescence parameters are closely related to chlorophyll content and the photosynthetic light system, this suggests that SLs may repair damaged PSII under drought stress and reduce thermal damage in tobacco, thereby restoring photochemical activity.

Under drought stress, excessive reactive oxygen species (ROS) accumulate in plants, disrupting their ROS metabolism (del Rio et al., 2006). In conjunction with the production of NADPH and ATP, the reaction between NADPH oxidase and O_2_ in the endoplasmic reticulum generates a substantial amount of O_2_^-^, leading to lipid peroxidation of cell membraness (Begum et al., 2020). This leads to cell wall damage, ion leakage, reduced enzyme activity, and cellular dehydration, resulting in metabolic disorders (Suganami et al., 2020; Xing et al., 2023). As excessive drought disrupts the dynamic balance of ROS formation and elimination, it reduces the drought tolerance of plants (Begum et al., 2020; Hafez et al., 2020). The generation rate of O2− and the content of MDA in the two flue-cured tobacco varieties increased with the severity of drought stress; however, the response was more pronounced in the water-sensitive variety Y116. Following the application of SLs, significant improvements were observed. Under severe drought stress, the generation rate of O2− and the content of MDA in Q96 decreased by 33.6% and 26.2%, respectively, whereas Y116 exhibited reductions of 45.7% and 49.4%. Mansoor et al (Mansoor et al., 2024). demonstrated that SLs stimulate the synthesis and accumulation of antioxidant compounds in plants, effectively scavenging reactive oxygen species (ROS) and alleviating toxicity associated with their excess. SLs were also shown to reduce MDA levels during stress and mitigate damage from lipid peroxidation (Raja et al., 2023). This indicates that SLs enhanced the antioxidant capacity of tobacco under drought stress, reduced ROS and intracellular MDA levels, and alleviated damage to flue-cured tobacco.

Plant cells possess a specialized antioxidant enzyme system, including SOD, POD, and CAT, which eliminates reactive oxygen species and regulates drought resistance while protecting cell membranes (Yue et al., 2022; Gelaw et al., 2023). Upon initial stress exposure, plants typically significantly increased SOD, POD, and CAT activities to counteract excessive reactive oxygen species production. However, excessive drought stress impairs the antioxidant enzyme system, diminishing its activity (Li et al., 2018; Gelaw et al., 2023). The enzyme activity of Q96 was significantly higher than that of the control under drought treatments D1 and D2, but significantly lower under treatment D3. In contrast, the enzyme activity of Y116 was significantly lower than that of the control under all treatments. Spraying exogenous SLs significantly improved the antioxidant enzyme activity of the two tobacco seedling varieties, although this enhancement diminished with increasing drought severity. In SLs-treated tomatoes, AsA and GSH levels were elevated, and antioxidant enzyme activities were increased to mitigate Cd toxicity (Raja et al., 2023). Exogenous SLs significantly increased antioxidant enzyme levels in tobacco under drought stress, thereby enhancing its environmental tolerance and drought resistance.

Osmoregulatory substances, which are fundamental metabolic products of plant cells, play a crucial role in regulating osmotic pressure and are correlated with plant life strategies (Suganami et al., 2020). They serve dual roles: regulating plant stress while safeguarding essential survival activities, and providing crucial energy reserves and metabolic information molecules that modulate the transcription levels of drought-resistant plant genomes (Varshney and Gutjahr, 2023). The proline and soluble sugar contents in Q96 and Y116 exhibited an initial increase followed by a decrease in response to increasing drought conditions. Our findings indicate that the application of exogenous SLs significantly elevated the levels of these two substances, enhancing stress resistance through improved osmotic adjustment. Previous studies have demonstrated that SLs play a crucial role in maintaining the osmotic balance of plant cells by promoting the expression of osmoregulation-related genes (Mostofa et al., 2018). Additionally, SLs regulate the sugar levels in plants, enabling them to optimize their production potential even under adverse conditions (Wang et al., 2020a).

The activity and gene expression of carbon and nitrogen metabolism enzymes serve as determinants for photosynthetic efficiency and carbohydrate accumulation in plants (del Rio et al., 2006; Iqbal et al., 2015). Our findings revealed a significant reduction in NR and Rubisco activities in the leaves of two tobacco varieties under drought stress. However, the application of SLs under drought conditions led to a significant increase in NR and Rubisco activities in the leaves of these tobacco varieties. It has been suggested that under soil nutrient deficiencies (e.g., nitrogen and phosphorus) or drought conditions, SL levels may increase, modulating plant root architecture and enhancing nutrient uptake across various adversities (Seto et al., 2019; Bürger and Chory, 2020; Kodama et al., 2022; Waadt et al., 2022).

Plant endogenous hormones, which are essential for signaling and regulation, respond to various stresses and are involved in multiple biological processes (Yao et al., 2019; Xia et al., 2023). These hormones typically maintain a specific equilibrium that facilitates normal plant metabolism and growth. However, under environmental stress, plants modify their physiological mechanisms and growth patterns by altering hormone concentrations (Azoulay-Shemer et al., 2015; Gelaw et al., 2023; Xia et al., 2023). Our findings indicate that drought stress significantly reduced the contents of IAA, GA, and ZR in the leaves of two tobacco varieties, while concurrently increasing ABA levels. Q96 exhibited significantly lower levels of endogenous hormones compared to Y116, suggesting that drought stress disrupts the hormonal balance in tobacco. This disruption weakens cellular metabolic activity, decreases cell water content, and promotes shedding and dormancy, ultimately impeding plant growth and development (Yao et al., 2019). The application of SLs enhanced the biosynthesis of IAA, GA, and ZR, while suppressing ABA formation. These results indicate that exogenous SLs can elevate endogenous hormone levels in plants under drought stress, thereby regulating leaf hormone metabolism. This regulation delays plant senescence, enhances photosynthetic performance, increases carbon assimilation, and mitigates drought-induced damage (Azoulay-Shemer et al., 2015; Yao et al., 2019; Yue et al., 2022; Xia et al., 2023). Although SLs have been well characterized as responsive to abiotic stresses in a coordinated manner through interactions with other hormones (e.g., SLs interact with ABA or IAA to regulate plant growth and development) (Mostofa et al., 2018), the mechanisms of these interactions are complex and varied, involving feedback regulation. However, due to the numerous types of hormones, the mechanisms by which SLs interact with other hormones are complex and varied (Wang et al., 2020b), involving feedback regulation, which cannot be simply summarized. To investigate how SLs influence the resistance of tobacco under drought stress, further examination of the expression of related genes is necessary.

This study conducted a detailed analysis of the physiological indices of two tobacco varieties, Q96 and Y116, under varying drought stress conditions and different SLs treatments, utilizing affiliation function calculations and principal component analysis (PCA) to identify key indices related to drought tolerance. The results indicated that the drought tolerance of Q96 and Y116 under various SLs treatments was primarily influenced by agronomic traits and photosynthesis, with a lesser impact from tobacco antioxidant enzyme systems and osmoregulatory mechanisms. Given that this study investigated 29 potential indicators related to drought tolerance in tobacco, the volume of data was substantial. Thus, employing PCA facilitated the identification of indicators that strongly represent drought tolerance. Additionally, the D-value, calculated from the affiliation function and weighted by PCA results, allowed for clearer comparisons. Ultimately, both tobacco varieties significantly mitigated the negative effects of SLs treatments applied under T1 and T2 conditions, corresponding to mild and moderate drought stress. The response to SLs treatments was more pronounced in Y116 compared to Q96, which exhibited greater differences in drought stress responses under T1 and CK conditions compared to D3. Y116 demonstrated significant differences from D3 under T1, T2, and CK treatments. Furthermore, we hypothesized that SLs enhance drought tolerance in tobacco through various pathways and mechanisms. Rather than merely exerting a one-sided positive effect, SLs function as a sustainable factor that contributes holistically to the initiation and development of a drought-tolerant system in tobacco.

In addition, we conducted a comprehensive evaluation and classification of the drought tolerance traits of two tobacco varieties, Q96 and Y116, using cluster analysis, Pearson correlation analysis, and principal component analysis. The results indicated that 19 traits (e.g., plant height, maximum leaf length, maximum leaf width, etc.) in Cluster 1 were significantly and positively correlated with drought tolerance in tobacco, which could serve as key indicators for improving drought tolerance in tobacco. In contrast, five traits in Cluster 3 (Ci, NPQ, O2− generation rate, MDA content, and ABA content) were negatively correlated with drought tolerance in tobacco, with an increase in these indices indicating a reduction in drought tolerance. The high correlation among traits within the same cluster suggests that these traits may share similar drought resistance mechanisms. Therefore, the inter-correlation of traits within the same cluster may simplify the identification of key indicators and enhance the efficiency of drought resistance trait assessment in future studies.

## 5 Conclusion

In this study, we investigated the effects of exogenous spraying of gibberellin lactone (SLs) on agronomic traits, the photosynthetic fluorescence system, reactive oxygen species metabolism, the antioxidant enzyme system, osmoregulatory substances, and hormonal regulation in two varieties, Q96 and Y116, to improve drought tolerance in flue-cured tobacco. Principal component analysis and affiliation function analysis were employed to evaluate the effects of spraying SLs, both with and without SLs, under various drought stress conditions on the drought tolerance of tobacco. The results indicated that the application of SLs effectively mitigated the effects of drought stress on flue-cured tobacco across various levels of drought, particularly in the water-sensitive Y116 variety. We also identified 16 drought tolerance traits that significantly influence drought tolerance in tobacco (Plant height、Maximum leaf length、Maximum leaf width、Chlorophyll a content、Chlorophyll b content、Tr、Gs、Ci、Fv/Fm、PSII、qP、SOD activity、POD activity、CAT activity、Pro、soluble sugar content). The identification of drought-related indicators in tobacco was simplified through cluster and correlation analyses, which categorized the traits based on their positive or negative correlation with drought tolerance in tobacco. 0.2 mg•L⁻¹ SLs enhanced the growth performance of flue-cured tobacco under drought stress, possibly due to the up-regulation of genes associated with the photosynthetic system, the antioxidant enzyme system and osmotic control system. SLs reduced the adverse damage experienced by both tobacco varieties under drought stress, thereby optimizing their growth potential. However, the interactions between exogenous SLs and various hormones (IAA, GA, ZR, and ABA) under drought stress remain unclear, and the associated molecular mechanisms require further investigation. This study demonstrated that SLs enhance drought tolerance in flue-cured tobacco through multiple pathways, serving as a sustainable and efficient approach to mitigate drought stress damage. Furthermore, the application protocol for SLs should be further explored to optimize the yield and quality of flue-cured tobacco.

## Data Availability

The original contributions presented in the study are included in the article/supplementary material. Further inquiries can be directed to the corresponding author.
